# Widespread Behavioral Responses by Mammals and Fish to Zoo Visitors Highlight Differences between Individual Animals

**DOI:** 10.3390/ani10112108

**Published:** 2020-11-13

**Authors:** Sarah A. Boyle, Nathan Berry, Jessica Cayton, Sarah Ferguson, Allesondra Gilgan, Adiha Khan, Hannah Lam, Stephen Leavelle, Isabelle Mulder, Rachel Myers, Amber Owens, Jennifer Park, Iqra Siddiq, Morgan Slevin, Taylor Weidow, Alex J. Yu, Steve Reichling

**Affiliations:** 1 Department of Biology, Rhodes College, 2000 North Parkway, Memphis, TN 38112, USA; nathanberry5@hotmail.com (N.B.); jac7pv@mail.missouri.edu (J.C.); fergusonsarah4@gmail.com (S.F.); allegilgan@gmail.com (A.G.); adikhandds@gmail.com (A.K.); hankailam@gmail.com (H.L.); stephen.leavelle@gmail.com (S.L.); isabellemulder0@gmail.com (I.M.); rmyers1116@gmail.com (R.M.); amber.r.owens@gmail.com (A.O.); jhp91@georgeotwn.edu (J.P.); iqrasohailsiddiq@gmail.com (I.S.); slemc425@gmail.com (M.S.); taylorweidow@gmail.com (T.W.); alex.j.yu@outlook.com (A.J.Y.); 2 Conservation and Research Department, Memphis Zoo, 2000 Prentiss Place, Memphis, TN 38112, USA; sreichling@memphiszoo.org

**Keywords:** exhibit, interactive, management, visitor effect, welfare, zoo

## Abstract

**Simple Summary:**

It is important to understand the impacts that humans have on zoo animals to ensure that zoo animal welfare is not compromised. We conducted multiple short-term studies of the impact of zoo visitors on 16 animal species and found that 90.9% of the mammal species and 60.0% of the fish species studied exhibited some change in behavior related to zoo visitors. Animals with behavioral changes were housed in exhibits with no direct contact with humans and exhibits with direct contact. These changes in behaviors were not always consistent across species, and often individual animals of the same species and living within the same exhibit had varied behavioral responses. We recommend (1) using short-term assessments to identify behavioral responses that may be of concern; (2) monitoring individual responses of zoo animals to humans; and (3) creating refuges where animals may choose to retreat.

**Abstract:**

The impact that humans have on zoo animals can vary based on the species of animal, exhibit design, and individual differences in behavioral responses. We independently analyzed data from 10 never-published studies that examined the impact of zoo visitors on zoo animal behavior. Of the 16 species studied, 90.9% of the mammal species and 60.0% of the fish species demonstrated a change in at least one behavior based on zoo visitor abundance or visitor behavior (e.g., noise, solicitation of interactions from zoo animals). In addition, behavioral changes associated with zoo visitors were present in animals housed in exhibits where there was direct contact with zoo visitors, as well as in exhibits where there was indirect contact and no direct contact. Individuals often varied in their behavioral responses, and some individuals appeared to seek out interactions with visitors. Our findings demonstrate that short-term research projects can provide valuable insight into individual animal-level and species-level responses to visitor abundance and visitor behavior in the zoo setting. We recommend that behavioral assessments focus on the analysis of behaviors of individual animals whenever possible, and we recommend that exhibits provide areas that allow for animals to retreat from the public view.

## 1. Introduction

Zoos can play important roles in conservation education, and the experiences that visitors have at a zoo can influence visitors’ perceived connections with the animals, as well as visitors’ responses to conservation messages [1,2]. Zoo visitors often rate their visits as positive when they can see the animals at close proximity [3,4]. Furthermore, visitors often show more interest in animals that are active [5,6], and some visitors report having positive emotional responses after having a direct interaction with a zoo animal [7]. However, popular zoo species can experience greater levels of noise from visitors, as larger crowds can lead to increased ambient sound [8]. As a result of factors related to zoo visitors (e.g., increased visitor abundance, increased noise), some animals have been shown to exhibit behavioral and physiological changes that can be indicative of stress [9,10]. 

Although studies on the impacts of human visitors on zoo animal behavior have existed for decades [11,12], documented responses of zoo animals to visitors have been inconsistent, and such variation is likely due to a range of factors (e.g., species studied, the animals’ individual personalities, characteristics of the zoo visitors, variability in exhibit design [13,14]). Furthermore, there is not full agreement on whether certain interactions between the visitor and zoo animal are positive, neutral, or negative [15]. Studies have examined a range of variables, from visitor abundance, density, and proximity [16,17], to visitor noise and visitor activity levels [8,18]. Additional studies are needed to understand the extent visitors impact the welfare of the zoo animals [19], as behavioral changes in an animal do not necessarily indicate a negative effect on animal welfare [13,20].

To date, most zoo visitor studies have focused on primates and felids [21]. Primates sometimes respond to zoo visitors [12,17], and at times the primates’ behavioral responses appear to signal that the animals are stressed by the zoo visitors [22,23]. Markers of behavioral stress include behavioral changes [11,24,25], differences in visibility while on exhibit [26], and elevated levels of glucocorticoids [27,28]. Nevertheless, it is difficult to make generalizable statements about the impacts of zoo visitors on primates. For example, Kuhar [26] found that overall western lowland gorillas (*Gorilla gorilla gorilla*) did not exhibit much behavioral change as visitor abundance increased; however, when crowd size was large, aggression increased in a bachelor male group, but not in a family group. Carder and Semple [29] noted a visitor effect in gorillas at one zoo, but not at another. Stoinski et al. [30] documented that overall gorilla behavior did not appear to be impacted by visitor abundance, but differences existed in the responses by individual gorillas. However, a study of chimpanzees (*Pan troglogytes*) and gorillas found that overall the animals’ behaviors and exhibit use did not vary with the number of visitors [31].

Similar variation also exists in felids. Margulis et al. [5] did not find evidence that visitor presence impacted six cat species: lion (*Panthera leo*), Amur leopard (*P*. *pardus orientalis*), Amur tiger (*P*. *tigris altaica*), snow leopard (*P*. *uncia*), clouded leopard (*Neofelis nebulosi*), and fishing cat (*Felis viverrinus*). However, Suárez et al. [32] noted behavioral differences based on the presence or absence of zoo visitors in five felid species: Eurasian lynx (*Lynx lynx*), jaguar (*P. onca*), bobcat (*L. rufus*), ocelot (*Leopardus pardalis*), and Asiatic lion (*P. l. persica*). A separate study of jaguars found that visitor density and noise level impacted the amount of time the cats spent visible and the male’s level of aggression, while visitor noise levels were associated with increased pacing by the female [33]. Therefore, even with the most-studied taxa (primates and felids), behavioral responses vary. 

Other taxa also show a range of responses. Zoo visitors had little to no impact in studies of flamingos (*Phoenicopterus roseus*, *P*. *ruber*, *P*. *chilensis*, *P*. *andinus*, and *P*. *minor* [34]), greater rheas (*Rhea americana* [35]), slender-tailed meerkats (*Suricata suricatta* [36]), and African penguins (*Spheniscus demersus* [37]). However, zoo visitors impacted aggressive, huddling, and avoidance behaviors [38], as well as location within the exhibit area [39], in little penguins (*Eudyptula minor*), aggression and activity levels in female Galápagos giant tortoise (*Chelonoidis nigra* [40]), and vigilance in koalas (*Phascolarctos cinereus* [41]). Furthermore, studies have found variable patterns related to inactive behavior: from resting more when visitor abundance was low [25] to resting more when visitor abundance was high [42], to no change in resting behavior [39]. 

Additionally, some zoo exhibits encourage direct physical interactions between the visitor and animals [43,44]. In visitor-feeding experiences with crowned lemurs (*Eulemur coronatus* [44]) and giraffe (*Giraffa camelopardalis* [45,46]), zoo animals did not appear to be negatively impacted by the increased visitor contact. Although petting-zoo goats (*Capra hircus*), llamas (*Llama glama*), and Vietnamese pot-bellied pigs (*Sus scrofa*) did not respond to zoo visitors in the same manner, Farrand et al. [47] concluded that the animals’ welfare was not negatively impacted by visitor interactions. Petting-zoo African pygmy goats (*Capra hircus*) and Romanov sheep (*Ovis aries*) showed a reduction in undesirable behaviors when a retreat area was available, underlying the importance of exhibit design in animal welfare [48]. When multiple exhibit types were studied, Bennett’s wallabies (*Macropus rufogriseus*) fed more and exhibited more interactive behaviors in the no-interaction exhibits; however, resting, locomotion, and vigilance did not differ between exhibit types [49]. In addition, quokka (*Setonix brachyurus*) were less likely to be visible when visitors were present in walk-though exhibits, but the effect of visitor presence was not considered to be great [50]. 

Individual animals can vary in their responses to zoo visitors [21,51,52]. A study of three polar bears (*Ursus maritimus*) found one animal increased and two animals decreased stereotypical behavior during periods of higher visitor density [53]. A recent review [21] highlighted that individuals may not perceive their environment in the same manner, even when these individuals are in the same exhibit; thus, individual traits and temperament may greatly impact behavioral responses. For example, the social rank of a Japanese macaque (*Macaca fuscata*) predicted probability of aggression towards zoo visitors [54]. Therefore, it is important to also consider individual differences when assessing zoo animal behavior [26] and welfare [55].

Although the behavior of some zoo animals may change when visitors are present, such behavioral changes are not necessarily negative: in a study of 12 primate species, the primates spent more time in the front of the exhibit and attempted to interact with visitors when large, active visitor groups were present [12]. When orangutans were given a preference test, they did not show aversion to the public viewing area [56]. When treatments were applied that varied in the extent of interactions that servals (*Leptailurus serval*) had with zoo visitors, the overall decrease in stereotypic pacing led to the conclusion that some visitor-encounter programs may have a short-term positive benefit for the animals [57]. Furthermore, the relationship between variables (e.g., visitor abundance, zoo animal behavior) is not necessarily causal: an increase in certain behaviors may attract zoo visitors [23]. Therefore, visitors and zoo animals may mutually impact the other’s behavior [5].

Here we present findings from 10 independent studies on 16 species that all focused on the impact of human visitors on zoo animals. Each of these 10 studies examined the extent to which one or more independent variable (e.g., abundance, noise, proximity, solicitation of interactions with zoo animals) impacted zoo animal behavior. Because individual animals may have different behavioral responses, whenever possible, we focused our analyses on individual animals, so that animals that demonstrated a change in behavior could be immediately identified, which is important for managing individual animals in a zoo [58,59]. Based on the findings from previous studies (summarized above), we predicted that zoo visitors would be associated with changes in interactive, vigilance, social, aggression, stereotypic, rest, and visibility behaviors of the zoo animals. 

Our work contributes new species to the body of literature on the zoo visitor effect: 10 of the 16 species studied were not represented in a recent review of the literature on zoo visitor impacts on animal behavior [21]. Four of these newly studied species were fish, on which there have never been published studies documenting their responses to zoo visitors [21], even though fish (e.g., Chondrichthyes) are often part of animal–visitor interactive exhibits [60]. Findings from research on how visitors affect zoo animals can be integrated into plans for exhibit designs, and hopefully create experiences that are positive for both the visitor and the zoo animal [61]. However, without published data for a wider representation of taxa at zoological parks, analyzing taxonomic-wide patterns of the impacts of zoo visitors on zoo animals is not fully possible. Based on findings from our studies, we make recommendations for zoo animal management, specifically in the context of the responses of zoo animals to humans.

## 2. Materials and Methods 

### 2.1. Study Subjects and Exhibit Design

Behavioral data were collected on 128 individual zoo animals representing 16 species of mammals and fish (Table 1; Appendix A) in 15 exhibits at the Memphis Zoo in Memphis, TN, USA. Data were collected during the months September and October 2010–2018, with each species studied once during a period of 4–5 weeks, with the exception of cownose ray and two groups of northern white-cheeked gibbons, all of which were studied twice in two different years. All studies were observational studies, and therefore did not require formal Institutional Animal Care and Use Committee (IACUC) approval. None of the data presented in this manuscript have been previously published.

Mammals were studied from 14 different exhibits, and fish from one multi-species exhibit. Eight mammal exhibits were outdoor, five were indoor, and one exhibit had both indoor and outdoor sections that were visible to the public (Table 1). There were no species which had one group housed in an outdoor public exhibit and another group housed in an indoor public exhibit. All exhibits contained enrichment items for the animals. Data collection did not occur during daily “keeper chats” where animals were often provided additional enrichment. The only studies that involved enrichment items directly in the research design were the studies of the fish, which were part of a touch tank and where zoo visitors could provide food to the animals.

The extent to which each exhibit encouraged interactions between humans and zoo animals varied. Each exhibit was categorized as: (1) traditional zoo exhibit design (*n* = 6): visitors were separated from zoo animals by a moat, water body, landscaping, or fences; (2) indirect contact design (*n* = 8): exhibit allowed non-direct interactions between zoo animals and visitors (e.g., glass window was a physical barrier between the zoo animals and visitors); or (3) direct contact design (*n* = 1): exhibit allowed direct interactions between zoo animals and visitors, allowing for physical contact (e.g., touching or feeding animals; Table 1). The only exhibit that allowed for direct contact was the interactive touch pool (Stingray Bay) where all the fish studies were conducted.

Most studies research one species at a time, and when meta-analyses are conducted, variation can be large [14]. Furthermore, there can be difficulties with small sample sizes, or confounds related to seasonal variation [26,62]. Our study examined a range of species during the same time of year at the same geographic location, thereby minimizing seasonal changes in behavior. 

### 2.2. Data Collection and Analysis

Each of the 10 studies represented an independent study to test to what extent visitors impact the behavior of zoo animals. All 15 researchers were trained and mentored by the same researcher from 2010 to 2018, and all studies used instantaneous scan-sampling methods [63]. Because each study was independent from all other studies, each study involved behavioral data collection by only one or two researchers; when two researchers worked together, one person collected scan sampling data for a species at a particular time. Scan sampling occurred every minute or every two minutes, depending on the specific characteristics of each study (e.g., number of individuals sampled, size of exhibit, species-specific characteristics such as quickness of movement across exhibit; details available in Appendix A). Such time intervals typically result in the same estimates for behaviors [64,65]; therefore, it was not a concern that our 10 studies used a mixture of one-minute or two-minute scan intervals. A typical observation period lasted 120 min (Appendix A). During each scan, the variables associated with human presence (Table 2) and the behavior of each animal in sight (Table 3) were recorded. 

For most of the 16 species, the ethogram (Table 3) was consistent across the studies. Because there were some species-specific behaviors (e.g., swimming was included only for the fish), full details for each of the 10 studies are provided (Appendix A Methods). We focused on the behaviors listed in the ethogram (Table 3) because these behaviors have been often analyzed in previous studies on the effect of zoo visitors on zoo animals (see [21] and the introduction of the current manuscript for a review). 

For three studies (Study IDs 7, 8, and 10) representing seven species (white-cheeked gibbon, Sumatran orangutan, cownose ray, southern stingray, bonnethead shark, white-spotted bamboo shark, and brownbanded bamboo shark), the physical locations of the zoo animals were also noted at each scan (details provided Appendix A Methods).

When possible, behavioral scans were conducted on individual animals, based on an individual’s recognizable physical characteristics. Such single-subject analyses can be important because individuals can have varied responses to variables [59]. When individual identities could not be confirmed throughout a study’s duration, group scan sampling was used to record overall group behavior on an interval. Although group sampling does not provide the detailed level of individual behavior that individual scan sampling provides, this methodology is reliable in studies of free-ranging animals in their natural habitat [66]. 

Each of the species in the 10 studies were analyzed separately because these 10 studies were fully independent of each other. We tested for the presence of a relationship between a human variable (e.g., visitor presence, visitor abundance, visitor proximity, or noise level; Table 2) and zoo animal behavior (Table 3) for each study independently (full explanation of statistical analyses are provided in Appendix A). Most studies addressed one independent variable (Table 2), unless stated otherwise.

Changes in such behaviors do not necessarily indicate a positive or negative welfare status [21]; therefore, our analyses focused on whether or not the human-associated variable was associated with changes in the zoo animals’ behaviors, and to what extent the behavior changed. We defined a change in behavior occurred when *p* ≤ 0.05 or *p* ≤ 0.025, based on the number of comparisons made, and we ran Bonferroni-corrected post-hoc tests to determine patterns in behavioral changes (please see the details in Appendix A). Whenever possible, the analyses focused on each individual animal, instead of overall patterns by the group of individuals. We chose this individual-based approach because zoo management plans that focus on individuals, instead of generalized responses by a population, can help focus on the well-being of each individual zoo animal [58].

No statistical analyses focused on identifying predictive variables indicating the likelihood that certain species would respond to humans. Such predictive variables could not be identified because (1) behavioral changes were noted in a large majority of species; and (2) confounding variables existed: for example, the fish were the only species in an exhibit where direct contact with zoo visitors occurred.

## 3. Results

### 3.1. Across All Taxa

**Figure 1:**
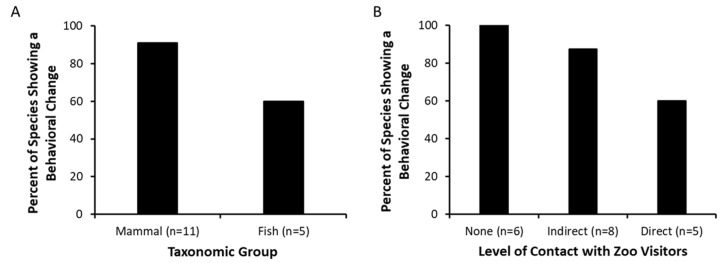
Behavioral changes associated with the presence of zoo visitors occurred in 13 of 16 species, including (**A**) a majority of the mammal and fish species studied; and (**B**) representing animals housed in exhibits that allowed for no contact with zoo visitors to direct contact with visitors (more than 16 species are noted because some species were in multiple exhibits).

Of the 16 species studied, 81.3% (*n* = 13) exhibited a change in at least one behavior related to the presence of humans. These behavioral changes occurred across taxa: 90.9% of the mammal species (grey wolf, cheetah, lion, Garnett’s greater galago, northern night monkey, Mona monkey, northern white-cheeked gibbon, bonobo, western lowland gorilla, and Sumatran orangutan) and 60% of the fish species (cownose ray, southern stingray, and bonnethead shark) exhibited some changes (Figure 1A). Behavioral changes were not documented in three species: southern tamandua and the two species of nocturnal bamboo sharks. Although variables were kept as constant as possible across the 10 independent studies, species differed in their expression of behaviors of focus (Table 3; detailed results of statistical analyses for behaviors exhibited for ≥5% of the scans are noted in Table S1 and Figures S1–S6). Aggressive behavior rarely occurred (<5% of scans).

Behavioral changes occurred in species living in exhibits that differed in the level of contact with zoo visitors (Figure 1B). These descriptive results are provided to demonstrate the range of species that showed a behavioral change in the studies; however, these species and exhibit characteristics are not necessarily independent of each other (e.g., all fish species were in a direct-contact exhibit, while none of the mammals were).

While individuals within a species (e.g., lion, cheetah, orangutan) had similar behavioral responses to zoo visitors, differences in individual responses occurred in other species (e.g., wolf, galago; Table 4). These differences were minor (gibbons, study ID 5: one behavior for one individual did not follow the pattern) to relatively complex (galagos: individual differences with four behaviors). In the gorillas, females showed more interactive behavior with humans when visitor abundance was high. Such behavior was greatest in the male when visitor abundance was low; however, the male engaged in interactive behavior twice as often as the females. 

### 3.2. Mammals: Visitor Abundance, Presence, Noise, and Solicitation of Interactions

Behavioral changes occurred in 90.9% of the mammal species studied (Table S1). The response of zoo animals to visitor abundance was studied in eight species, and all eight species (wolf, cheetah, lion, night monkey, mona monkey, white-cheeked gibbon, bonobo, and gorilla) demonstrated behavioral changes to some extent. For most species, as zoo visitor abundance increased, interactive, vigilance, and alert behaviors increased, and social, rest, and out-of-sight behaviors decreased (Table 4). However, these patterns did not hold for all species (e.g., social increased in night monkeys with greater visitor abundance; rest decreased in bonobos when visitor abundance low). Although individual responses were consistent in the cheetahs, lions, and most of the gibbons, for some behaviors there were differences in the responses by individual wolves (alert and rest; Figure S1), and between the male gorilla and three female gorillas (interactive and rest; Figure S3).

There were two groups of gibbons, and both groups were studied twice (two different years). The group of gibbons housed in the indirect-contact exhibit, where visitors and gibbons could interact through a set of long windows, consistently demonstrated behavioral changes based on the abundance (ID: 5; Figure S3) and presence (ID: 7; Figure S4) of visitors. In both studies, as visitor abundance increased or as visitors were present, the gibbons increased their interactive behaviors (e.g., placing hands on window, making eye contact), but decreased their conspecific social and rest behaviors. These patterns held overall for the other group of gibbons housed in a traditional exhibit during one study (ID: 5; Figure S3), but not for the second study (ID: 6; Figure S5). Such findings highlight the importance of studying animals at different time points, as variables may change over time (e.g., Study IDs 5 and 6 differed because the son of the mated pair was transferred to another zoo). However, Study ID 6 did not examine behavior for each individual gibbon. Therefore, it is likely that data collection methods may greatly impact one’s conclusions: individual scan sampling occurred for all of the gibbon studies, with the exception of ID 6, which employed group scan sampling methods. As seen in several species (Table 4), often there were individual differences in behavior. Such differences between individuals can be muted when data are collected via group scan sampling. We acknowledge that these differences in results may be due to year-to-year changes in group composition, or due to differences in sampling methods. That said, we think it is important to highlight these factors because often studies address one social group for a short period of time, or studies may approach analyses from the level of only the group and not of individual animals. Our findings suggest that whenever possible, it is best to collect and analyze data on the level of the individual animal. 

Lastly, two studies examined the variable of visitor abundance or presence one step further, by categorizing the human behaviors. The wolf study categorized visitor noise level and found that three of the four wolves exhibited a behavioral change, with two wolves having increased alert behavior when visitor noise was at the greatest level (Figure S1). These findings suggest that crowd size and/or noise may be important factors in behavioral responses by some animals. In one of the gibbon studies (ID: 7), visitor presence increased gibbon interactive behavior, increased the percent of time that gibbons were located at the exhibit window, and decreased conspecific social and rest behaviors; however, no behavioral differences were detected when comparing situations when visitors were present and soliciting interactions from the gibbons (e.g., touching, tapping, or banging on the windows) versus visitors were present and not attempting to engage with the gibbons (Figure S4). These findings suggest that the gibbons did not avoid the visitors when the humans were soliciting engagement. 

### 3.3. Mammals: Human Proximity

Proximity between humans and zoo animals was studied in two studies (Study IDs: 4 and 8). The first study analyzed behaviors based on the proximity of the human to the exhibit window of the galagos (3 exhibits) and tamanduas (1 exhibit). All four galagos demonstrated some change in behavior across three levels of human proximity (close, medium, far), but the behaviors that changed (e.g., interactive, rest, stereotypic, and out of sight) varied for the four individuals (Table 4; Figure S2). For example, two individuals exhibited stereotypic behavior for ≥5% of the scans (5–6% of scans for both individuals), but one galago increased stereotypic behavior when humans were at the exhibit window, and the other increased stereotypic behavior when humans were within 1 m of the window. These two individuals were housed separately: one with another galago that exhibited stereotypic behavior for < 1% of the scans; the other galago was not housed with a conspecific.

Neither tamandua differed in their behaviors based on visitor proximity. The male exhibited stereotypic behavior for 61.8% of the behavioral scans (Figure S2), and the stereotypic behavior did not change based on the proximity of the human. The female, however, exhibited minimal stereotypic behavior (0.32% of scans). Both the female and male were out of sight for a relatively large amount of time (38.8% and 20.8%, respectively), in comparison with the other species in this study. 

While the galago and tamandua proximity study examined behavioral responses based on human proximity to the exhibit window, the orangutan proximity study focused on determining if the proximity of the human impacted the physical location of the orangutan, and if orangutans showed behavioral differences when the human and orangutan were in closest proximity. One of the two female orangutans was in closest proximity with the human observer more often than by chance; the second female did not show such a pattern. In fact, this female’s behavior closely approached statistical significance for avoiding the human observer (Table S1). When the females were in closest proximity to the human, both engaged in interactive behavior more and rested less (Figure S5). There were individual differences in social behavior; the female who spent little time in closest proximity with the human engaged in more social behavior when in closest proximity with the human. This finding is likely because the other female, with whom she was social, was in closest proximity with the human more often than by chance.

### 3.4. Fish: Visitor Abundance, Presence, and Food Provisioning

Cownose ray, southern stingray, and bonnethead shark all exhibited some extent of behavioral differences based on human behavior (Table 4). The variable visitor abundance was studied for only one species, the cownose ray (Study ID 9). The cownose ray decreased social behavior and increased solitary behavior as the abundance of visitors increased along the perimeter of the direct-contact touch pool (Figure S6A). 

In the analysis of the impacts of food provisioning on behavior and location within the exhibit (Study ID 10), southern stingray increased their swim and decreased their rest behaviors when visitors added food into the exhibit pool, and bonnethead sharks increased the percentage of scans they were located on the periphery of the exhibit when food was provisioned (Figure S6B); however, the southern stingray did not exhibit differences in their use of the periphery or inside of the pool, and bonnethead sharks did not exhibit differences in swim and rest behavior based on food provisioning (as swim behavior occurred more than 80% of the scans when food was provisioned and when no food was provided). The cownose ray did not exhibit differences in swim behavior (it remained more than 97% of scans under the conditions of food provisioned and food not provisioned), nor in location. The two species of nocturnal sharks (brownbanded and white-spotted bamboo sharks) did not exhibit behavioral changes; they spent more than 85% of all scans resting in the inside section of the pool. Such findings were not surprising given that these sharks are nocturnal, but the exhibit was a diurnal-focused exhibit, located in a partially open-air tent.

## 4. Discussion

In 13 of the 16 species studied, we found evidence that visitors impact the behavior of zoo animals. Behavioral changes were noted across taxonomic groups, with all but one mammal species demonstrating a behavioral change associated with zoo visitors. Furthermore, we noted behavioral changes in animals housed in exhibits with no contact allowed between zoo visitors and zoo animals, exhibits allowing for indirect contact (e.g., glass window), and exhibits allowing for direct contact between zoo visitors and animals. We acknowledge that our 10 studies were independent studies that did not focus on the same independent variables for all studies, and that direct comparisons cannot be made across all 16 species as to what primarily influences behavioral changes in zoo animals. However, such comparisons were not the intent of our analysis. Instead, our goal was to document the extent to which a range of variables associated with zoo visitors were related to behavioral changes in zoo animals.

Overall, we found that as visitor intensity increased in abundance, noise, and/or proximity, a majority of the animals studied demonstrated an increase in alert, vigilance, or visitor-interactive behaviors, and a decrease in social and rest behaviors. However, patterns were not consistent across all species, and behavioral differences sometimes existed between individuals living within the same social group. Furthermore, zoo visitor abundance was not consistent across exhibits: both exhibits of white-cheeked gibbons attracted relatively large numbers of visitors (Appendix A), while the northern night monkeys never had more than seven visitors at one time, suggesting that that the visitor effect can potentially impact individuals in particular exhibits more frequently and for longer durations than individuals in exhibits that are not frequented by visitors as often and to the same extent. 

The results from our studies support previous findings that the responses to zoo visitors can be variable [13,14]. Zoo visitors had an impact on the behaviors for 81.3% of the species we studied, but the lack of a behavioral change noted for three (18.7%) of the species we studied does not lead us to conclude that these three species were not impacted by zoo visitors. First, in some of the studies, the researchers did not identify individual animals; therefore, potential differences in individual animals’ behavioral responses were not detectable in those studies. Second, the three species for which we did not detect behavioral changes (tamandua, brownbanded bamboo shark, and white-spotted bamboo shark) are primarily nocturnal. While one species, tamandua, was housed in an exhibit set for nocturnal conditions, the two species of bamboo sharks were in a diurnal exhibit. These bamboo sharks spent most of the study resting in one location that was not in close proximity to where human visitors stood. Third, our 10 studies rarely measured more than two zoo visitor variables at a time. It is possible that for studies where no behavioral change was detected, had the study examined additional variables, the results may have differed. For example, Choo [16] found that overall orangutans were impacted by the proximity of zoo visitors, but not by visitor abundance or activity. In our study, the wolves’ behaviors often changed as visitor abundance and visitor noise changed, but there were no differences based on visitor presence versus absence. Therefore, future studies that record both visitor abundance as well as visitor noise and proximity could help tease apart the factors that most prominently impact the zoo animals. Such information can then be used directly by the zoo in their communication with visitors. For example, Sherwen et al. [36] found that the use of signs and the positioning of zoo employees reduced visitor noise around a meerkat exhibit.

The goal of our study was to determine to what extent the zoo animals demonstrated behavioral changes associated with changes in zoo visitor abundance, presence, noise, proximity, or food provisioning. We acknowledge that individual animals may vary drastically in their previous experiences with zoo visitors (e.g., frequency, intensity, and overall nature of interactions with visitors). Further, individual animals can vary in how they cope with changing environments and stressful situations [21,54]. That said, given the dearth of information published on some of the species we studied, documenting the behavioral changes that occur is a first step in gaining a better understanding of the visitor effect, and understanding how individual animals respond. Our studies examined behavioral changes at the individual level, whenever possible, because of the importance of understanding individual animals’ responses [58,59].

Although some behaviors (e.g., stereotypic behavior, aggression) are often associated with negative animal welfare, and other behaviors (e.g., play, affiliative social interactions) are sometimes associated with positive welfare [40,67], Sherwen and Hemsworth [21] stressed that many behaviors may not have clear associations with welfare status, and behavioral changes do not always indicate that the change is negative [68]. We acknowledge that an animal’s behavioral response (for example, decreasing rest behavior) does not necessarily indicate a particular emotional response. Therefore, we did not categorize the behaviors based on what the welfare implications were. We present our findings to add to the body of scientific literature on the responses of animals to zoo visitors, to help form a better understanding of zoo animal responses.

Based on our findings from studies of 16 species, we discuss below our recommendations for the management of zoo animals. These recommendations address (1) the importance of short-term studies that allow for the assessment of behavioral responses by individual animals, (2) the extent to which exhibit design may impact individual animals, and (3) considerations for future research studies. 

### 4.1. Study Design: Short-Term Assessments on Individual Animals

We recommend that when it is not feasible to conduct long-term projects on many species in multiple exhibits at a zoo, short-term monitoring programs that are based on well-established, species-appropriate ethograms can provide a great deal of information on a range of species at a particular zoo, and across multiple institutions. The benefits of individual-based monitoring are known [58,59]. Such monitoring programs could involve animal keepers and zoo educational staff, as well as members of the public (e.g., trained zoo volunteers, students taking a behavior course). These short-term assessments can quickly highlight if there are species or individuals that may be of concern. 

Previous studies have demonstrated that individuals can vary in their behavioral responses [21,51,52]. We found further evidence that individual animals of the same species in the same exhibit do not always respond in the same manner to humans. Therefore, we also recommend that studies are designed so that behavioral responses by individual animals may be detected. Although it may be necessary at times to collect data on the entire group, such as when it is not possible to accurately identify individuals, we suggest that data on individual animals are collected whenever possible. Single-subject experimental designs (SSDs) can also be important [59], especially if there is an individual animal of concern. While it is important to gain a general understanding of the impact of visitors on all species at a zoo, individuals within a species may react in different ways to visitor presence and other variables associated with visitors [8,30]. Therefore, it may be worth doing these initial assessments, and then consider SSDs or more involved, long-term studies to examine if changes in exhibit design, visitor behavior, or management practices lead to changes in the impact on the zoo animals (in the case that the impact was originally deemed to be negative). 

### 4.2. Exhibit Design

Zoo visitors are often interested in seeing zoo animals in close proximity [4], but proximity to humans can be a source of stress for zoo animals [69]. Sometimes modifications to enclosures can minimize the visitor effect: when netting in front of an enclosure modified the visibility between gorillas and zoo visitors, the gorillas’ behaviors changed (reduced aggression and stereotypical behavior), but the visitors’ perceptions also changed [70]. In a separate study, when visual contact between zoo visitors and capuchins (*Sapajus apella*) was reduced, the capuchins exhibited a decrease in aggression and in fecal glucocorticoid metabolite concentration, but the number of visitors also decreased [28]. Therefore, having zoos monitor the visibility of the animals [71] can address the zoo visitors’ experiences as well as identify potential issues relating to animal stress.

We found widespread behavioral responses of animals to zoo visitors, and these responses occurred in animals in traditional exhibits to animals in exhibits that were designed to encourage interactions between zoo animals and visitors. All species (100%) that had more traditional exhibits (e.g., zoo animals and visitors were physically distanced from each other due to a water body, elevation difference, and/or vegetation) demonstrated some level of behavioral change associated with zoo visitors. Behavioral changes also occurred for the majority of species in indirect-contact and direct-contact exhibits (87.5% and 60% of species, respectively). These findings illustrate the widespread extent to which zoo animals respond to visitors. 

An effective exhibit design can help protect the animals from potential negative consequences of large numbers of visitors [16,31]. In some exhibits, animals rotate on and off exhibit; it is possible that having restricted access to on-exhibit areas (which are often outdoors) could impact the animal’s behavior, as could being off exhibit where zoo visitors are not present. Based on our findings, we recommend that attention is paid to how individuals use their exhibit, and we recommend that all exhibits offer areas of refuge that are adequate in size for all individuals to enter at one time. 

Although zoo animals may seek refuge at times, visitors can potentially be a source of environmental enrichment for the zoo animals [20,56]. In our study, when visitors were present at a window, the gibbons spent more time interacting with the humans than they did with each other, and the gibbons spent more time at the window when visitors were present. However, when visitors were present at the window, there was no difference in gibbon behavior when the visitors solicited interactions with the gibbons compared to when the visitors did not initiate contact. These findings suggest that the visitors attracted the attention of the gibbons, but whether or not it was the visitor who initiated contact did not influence the behavior of the gibbons. Because the gibbons were able to access the entire exhibit, but the interactive window was only available on one side of the exhibit, the gibbons had to approach the window to interact with the humans. Our findings appeared to indicate that the gibbons sought out interactions with the humans. The gibbons did not rest or engage in social behavior as often as they did when no visitors were present, but additional study is needed to determine to what extent visitors could potentially impact short- and long-term social bonds between conspecifics in the same social group. 

We found that some individuals appeared to initiate interactions with humans (e.g., the gibbons in the indirect-contact exhibit), not all animals did so. For example, the wolves’ behavioral changes (e.g., increased alert behavior and decreased rest behavior in some individuals) appeared to be a response to the visitor abundance and visitor noise. For both tamanduas we studied, no differences in behaviors were detected based on human proximity, but the sustained stereotypic pacing of the male tamandua and the frequency that both animals were on exhibit yet hidden from view are important findings to address overall in regards to the housing and husbandry of these individuals. These differences in responses could be due to species differences or each individual animal’s history with humans; a more extensive data set is needed to draw such conclusions. 

In our study of the orangutans, the location of the human observer appeared to impact the physical location of one of the female orangutans. Furthermore, for both female orangutans, they rested less and interacted with humans (e.g., direct eye contact, reaching toward a human) more when they were in closest proximity with a human observer. Although this study did not focus on quantifying zoo visitor abundance, it did provide novel findings about the potential impacts of human observers on zoo animals. We found the orangutans interacted with humans through gazing and gesturing behaviors, both of which are ways to communicate information between two individuals [72,73,74]. Whereas Kaplan and Rogers [73] found that captive orangutans avoided direct stares and exhibited less direct gazes in comparison to wild orangutans, the two female orangutans at the Memphis Zoo did directly look at humans. However, the responses of the two female orangutans were not identical, as one of the females spent much less time in close proximity with humans, and the male, when on exhibit, spent all of the time in one spot, elevated and at a distance from humans. Such varied responses to exhibit use and humans highlight the importance of assessing individual animals’ behaviors, and determining to what extent the exhibit’s features allow each individual to choose the extent to which they are in view of (or proximity to) humans. 

The only exhibit that allowed for direct contact between zoo visitors and zoo animals was the interactive fish exhibit. Behavioral changes were noted in the cownose ray, southern stingray, and bonnethead shark. Although cownose rays increased solitary swimming behavior (and decreased social swimming) when more visitors were present, food provisioning impacted neither the time spent swimming versus resting, nor the physical use of the pool (periphery versus inside). These findings suggest that there may be variability between the individual cownose rays (as the groups were not the same from year to year), or that the rays had different responses to visitor variables. For example, the abundance of visitors may impact the rays in a different manner than whether or not the visitors are engaged in feeding the rays at a particular time, as the rays may anticipate receiving food from the visitors. 

### 4.3. Recommendations for Future Studies

Visitors tend to show a greater interest in mammals, as well as animals with larger bodies and higher activity levels [6]. Furthermore, much of the published literature also focuses on mammals, with emphases on primates and felids [21,75]. Our study includes findings on understudied (or never-studied) species, which are first steps in adding to the general understanding of how zoo animals respond to humans. However, many unknowns still exist regarding the impacts that zoo visitors have on captive species. Ideally, we would be able to identify the primary variables associated with species (or individuals) that demonstrate behavioral changes associated with the visitor effect. However, determining these predictive variables is a difficult task, as the responses by the animals may be based on a variety of factors. Multiple variables (e.g., enclosure design, interactions with zoo public, proximity to potential predators, interactions with animal keepers) can impact the behavioral and physiological responses of zoo animals [11,76,77,78]. As the number of studies increase, and the number of species expands, identifying predictive variables may be more possible. We recommend that studies of the impacts of zoo visitors expand to include species that are underrepresented in the literature, and, when possible, take note of behaviors associated with individual animals.

Recently there has been an increase in research addressing the welfare of zoo animals [79], specifically research that measures behavior and physiology [80]. There is no single strategy for assessing welfare that is most appropriate for all zoo animals [81], as the needs and responses of these animals vary by species [82], as well as on an individual basis [59,83]. Furthermore, it may be that factors other than (or in addition to) zoo visitors are primarily impacting an animal’s behavior. For example, in our study the male tamandua spent 61.8% of the scans exhibiting stereotypic pacing, and such pacing was consistent throughout the study, at different levels of visitor presence. Our finding suggests that additional factors may have been contributing to the stereotypic behavior of this individual animal, but we cannot rule out that zoo visitors did not contribute to the pacing behavior. In such situations, we recommend a holistic approach to animal management to examine multiple factors that may be impacting a particular individual animal. 

Noise and disruptions may lead to stress in some zoo animals, resulting in physiological changes as well as behavioral changes [9,10,27,69]. In addition, minimizing such disruptions could be critical for targeted breeding programs of threatened or endangered species. Further research on the hormone profiles of zoo animals could provide a better understanding of both behavioral and physiological factors related to zoo visitor presence. Such information taken together would be helpful in then assessing whether any changes indicate an animal welfare concern [19]. 

## 5. Conclusions

We found that more than 80% of the species in our study indicated some degree of behavioral change related to the presence of zoo visitors. Furthermore, we documented behavioral changes in individuals housed in exhibits that vary in their exposure to zoo visitors (no contact to direct contact allowed). Our findings provide evidence that a range of individuals modified their behaviors; however, sometimes some individuals of a species within the same exhibit exhibited a behavioral change while other individuals did not. We recommend that the monitoring of zoo visitors’ impacts on zoo animals should be expanded to account for the variety of species housed in captivity, and to ensure that individual zoo animals are not responding in a manner that suggests that they are experiencing stress from the zoo visitors.

## Figures and Tables

**Table 1:** Animals studied and their exhibits. An asterisk (*) represents species for which we found no previous peer-reviewed scientific literature related to the impact of zoo visitors on the species.

Common Name	Species	Exhibit ^1^	Study ID ^2^
Grey wolf	*Canis lupus*	O; 1	1
Cheetah	*Acinonyx jubatus*	O; 1	2
Lion	*Panthera leo*	O; 1	3
Southern tamandua *	*Tamandua tetradactyla*	I; 2 ^3^	4
Garnett’s greater galago *	*Otolemur garnettii*	I; 2 ^3^	4
Mona monkey *	*Cercopithecus mona*	O; 1	5
Northern night monkey *	*Aotus trivirgatus*	I; 2 ^3^	5
Northern white-cheeked gibbon	*Nomascus leucogenys*	O; 1, 2 ^4^	5, 6, 7
Bonobo *	*Pan paniscus*	I/O; 2	5
Western lowland gorilla	*Gorilla gorilla gorilla*	O; 2	5
Sumatran orangutan	*Pongo abelii*	O; 1	8
Cownose ray *	*Rhinoptera bonasus*	SE; 3	9, 10
Southern stingray *	*Hypanus americanus*	SE; 3	10
Bonnethead shark *	*Sphyrna tiburo*	SE; 3	10
Brownbanded bamboo shark *	*Chiloscyllium punctatum*	SE; 3	10
White-spotted bamboo shark *	*Chiloscyllium plagiosum*	SE; 3	10

^1^ Exhibit descriptions include their exposure to the local environment (I = indoor; O = outdoor; I/O = indoor and outdoor sections; SE = semi-enclosed tented area), and the extent to which the exhibit was designed to encourage interactions with humans (1 = typical zoo exhibit design, i.e., visitors are separated from zoo animals by a moat, water body, landscaping, or fences; 2 = exhibit allows non-direct interactions between zoo animals and zoo visitors, i.e., indirect contact can be made via glass partition; 3 = exhibit allows direct interactions between zoo animals and zoo visitors, i.e., contact occurs). ^2^ The study identification number is used throughout the manuscript so that the studies may be quickly referenced. ^3^ Exhibit was in the nocturnal house, so exhibit was dark during data collection hours. ^4^ One exhibit was a typical zoo exhibit, while the other exhibit allowed for interactions through glass windows.

**Table 2:** Human-focused independent variables addressed in 16 species across 10 studies ^1^.

Variable	Definition	Species of Study ^2^
Abundance	Number of zoo visitors located in the public viewing area. Four categories: 0, 1–4, 5–9, or ≥10 visitors	Wolf, cheetah, lion, mona monkey, owl monkey, gibbon (ID: 5, 6), bonobo, gorilla
Abundance (aquatic) ^3^	Number of zoo visitors peering or reaching into the pool. Four categories: 0, 1–10, 11–20, and ≥ 21 visitors	Cownose ray (ID: 9)
Presence	Two categories: (1) 0 visitors and (2) ≥1 visitors present	Gibbon (ID: 7)
Noise	Three categories: (1) low (visitors walking, talking softly, or pushing a stroller); (2) medium (“low” plus running or talking loudly); (3) high (“medium” plus playing loud music, yelling, and/or howling)	Wolf
Solicit interaction	Two categories: (1) visitor present at the exhibit window, but is not initiating contact (not touching the window); (2) visitor present at the exhibit window, and is touching, tapping, or banging on the window	Gibbon (ID: 7)
Proximity (1)	Three categories: (1) close (at the exhibit’s glass window); (2) medium (<1 m from the window); and (3) far (1–2 m from window)	Tamandua, galago
Proximity (2)	Two categories: (1) human observer and study animal within closest proximity; (2) human observer and study animal at greater distance than first category	Orangutan
Food provisioned (aquatic)	Two categories: (1) visitor(s) put fish into pool; (2) no fish was added to the pool	Cownose ray, southern stingray, bonnethead shark, brownbanded bamboo, white-spotted bamboo shark (ID: 10)

^1^ Each study measured one or more of these human-focused variables. Most studies addressed only one human-focused variable. The human variable was recorded at each scan sample. Details are available in Appendix A. ^2^ For species that were studied multiple times, the study identification number (ID) is listed for clarification. ^3^ The classifications for aquatic visitor abundance were larger than for non-aquatic visitor abundance because the interactive pool allowed for visitors to position themselves around the entire perimeter of the pool. None of the non-aquatic exhibits allowed for visitors to be around the entire perimeter.

**Table 3:** Ethogram for the behaviors studied ^1^.

Behavior	General Definition
Mammals	
Interactive	Approach human with eyes on the human; approach the glass window or place appendages on window; make direct eye contact with or gesture towards human (Used only in primate and tamandua studies).
Vigilance	Prolonged stare at a specific location, following a human with eyes or head, or scanning the exhibit with eyes and head (Used only in felid studies: cheetah and lion).
Alert	Look in one direction while resting with head up, standing, walking, running, stalking, sitting upright, or vocalizing (Used only in wolf study).
Social	Interact with another conspecific individual through touch (e.g., grooming, huddling bodies).
Aggression	Agonistic interactions between individuals through physical contact.
Stereotypic	Repetitive movements (e.g., pacing).
Rest	Remain in one location without movement. Sitting or prone position.
Out of Sight	Animal confirmed to be on exhibit but hidden from sight of the human observer.
Fish	
Swim	Forward movement in the water.
Rest	No movement in the water.
Enrichment	Swim through or on top of, or rest within or on top of, enrichment item.
Social	Within two body lengths of one of more conspecifics; if swimming, swimming in the same direction.
Solitary	More than two body lengths from a conspecific; if swimming, more than two body lengths or swimming in the opposite direction.

^1^ Specific ethograms for each of the 10 independent studies of 16 species are provided in Appendix A. The behaviors listed here were all behaviors analyzed. These behaviors were part of the ethograms for multiple species, but these behaviors were not analyzed when the species exhibited them infrequently (<5% of behavioral scans). All ethogram behaviors were analyzed for at least one species, except for aggression. Researchers recorded when instances of aggression occurred, but these occurrences were so infrequent (or did not occur at all) that they did not comprise a sample size large enough for analysis for any of the species (or individuals).

**Table 4:** Summary of behavioral changes associated with zoo visitors ^1^.

Human Variable ^2^	Zoo Animal Behavior ^2^	Common Name (# of Individuals with Change)
Abundance↑	Interactive↑	Mona monkey ^3^; gibbon (3 of 4—ID: 5); gorilla (females) ^3^
	Vigilance↑	Cheetah (2 of 2); lion (3 of 3)
	Alert↑	Wolf (2 of 4)
	Social↓	Gibbon (3 of 4—study ID: 5) bonobo ^3^; cownose ray ^3^ (ID: 9)
	Social↑	Night monkey ^3^
	Rest↓	Lion (3 of 3); Mona monkey ^3^; night monkey ^3^; gibbon (4 of 4—ID: 5); gorilla (females) ^3^
	Rest↑	Bonobo ^3^
	Out of sight↓	Mona monkey ^3^
Abundance↓	Interactive↑	Gorilla (1 of 1 male) ^3^
Presence: Yes	Interactive↑	Gibbon (2 of 2—ID: 7)
	Rest↓	Gibbon (1 of 2—ID: 7)
	Social↓	Gibbon (2 of 2—ID: 7)
	Located at window↑	Gibbon (2 of 2—ID: 7)
Noise level↑	Alert↑	Wolf (2 of 4)
Proximity: Close	Interactive↑	Galago (1 of 4); orangutan (2 of 2)
	Rest↓	Galago (2 of 4); orangutan (2 of 2)
	Social↑	Orangutan (1 of 2)
	Out of sight↑	Galago (1 of 4)
	Out of sight↓	Galago (2 of 4)
Food provisioned	Rest↓/Swim↑	Southern stingray ^3^ (ID: 10)
	Periphery of exhibit↑	Bonnethead shark ^3^ (ID: 10)

^1^ Listing is only when behavioral differences occurred, and only for results where a clear pattern emerged (e.g., an increase in alert behavior when visitor abundance increased). A behavioral change was determined when *p* ≤ 0.05 or *p* ≤ 0.025, depending on the number of variables analyzed (Appendix A; Table S1). Patterns of the responses were determined based on Bonferroni-adjusted post-hoc tests (Figures S1–S6). For species where there were multiple studies, the study identification number (ID) is noted. A behavior was analyzed for an individual or species only if the behavior was represented in ≥5% of the behavioral scans. Details on the behaviors tested are in Appendix A. ^2^ The human variables are defined in Table 2, while the zoo animal behavior is defined in Table 3. ^3^ Results represent patterns from group scan sampling, not individual scan sampling.

## Supplementary Materials

The following are available online at https://www.mdpi.com/2076-2615/10/11/2108/s1, Table S1: Full summary of results, listed in order by Study ID 1. All zoo animal behaviors of interest (Table 3) that occurred ≥5% of the scans are noted. Items in bold indicate a significant difference between comparison groups of the independent variable (human variable), based on the adjusted alpha-level from Bonferroni corrections. Where differences existed, the direction of patterns in the differences are noted. Results from pairwise comparisons indicated a direction of difference (Figures S1–S6), Figure S1: Individual wolves demonstrated varied responses to visitors: In some of the wolves, (A) alert and (B) rest behavior differed with changes in visitor abundance, and (C) three of the four wolves showed changes in alert behavior as visitor noise increased. Cheetahs (D) increased vigilance behavior at higher levels of visitor abundance, but rest behavior did not change. Lions (F) increased vigilance behavior and (G) decreased rest behavior at higher levels of visitor abundance. Horizontal lines indicate a difference in behavior for an individual across the levels of the independent variable, with the p-value provided above the line. Results from post-hoc Bonferroni tests are noted by letters above the bars; different letters indicate a pairwise differences at an alpha-level of 0.008 (A, B, D, and E) and 0.017 (C, F, and G). Data represent findings for three studies: wolves (Study ID 1), cheetahs (Study ID 2), and lions (Study ID 3), Figure S2: Increased proximity of humans to the exhibit was associated with (A) increased interactive behavior in one of four galagos; (B) no change in social behavior in two galagos; (C) inconsistent changes in stereotypic behavior in two galagos, while the male tamandua consistently exhibited high levels of stereotypic behavior; (D) decreased resting behavior for two of four galagos; and (E) inconsistent behavioral changes in three of four galagos, with no pattern of behavior change in the two tamanduas. Horizontal lines indicate a difference in behavior for an individual across the levels of the independent variable, with the p-value provided above the line. Results from post-hoc Bonferroni tests are noted by letters above the bars; different letters indicate a pairwise differences at an alpha-level of 0.017. Data represent findings for Study ID 4, Figure S3: In one of the primate studies (Study ID 5), greater levels of visitor abundance were (A) associated with increased interactive behavior in the Mona monkeys, gibbons, and female gorillas; but (B) changes in social behavior did not follow a clear pattern across species. Increased visitor abundance was associated with (C) decreased rest in most of the primates, except for the bonobos and male gorilla, Mwelu. Only one species had more than 5% of scans out-of-sight, and such behavior was noted in the (D) Mona monkeys only when visitor abundance was low. An “X” indicates that the particular category for visitor abundance was not included in the analysis due to low sample size, while a “0” indicates that the behavior did not occur during the scans at that level of visitor abundance. There were always at least one visitor present for all scans, so there was no category of “0 visitors” in these analyses. Horizontal lines indicate a difference in behavior for an individual across the levels of the independent variable, with the p-value provided above the line. Results from post-hoc Bonferroni tests are noted by letters above the bars; different letters indicate a pairwise differences at an alpha-level of 0.017. Data represent findings for study ID 5, Figure S4: A sibling pair of northern white-cheeked gibbons was able to interact indirectly with zoo visitors via a long window that ran along the length of one side of the exhibit (Study ID 7). (A) When visitors were present, both the male and female gibbon exhibited increased interactive behavior, decreased social behavior with the other gibbon, and spent more time at the viewing window. The male also rested less when visitors were present. (B) When visitors were present at the window, there was no difference in behaviors by the gibbons when the visitors initiated contact with the gibbons versus when the visitors did not initiate contact. Horizontal lines indicate a difference in behavior for an individual across the levels of the independent variable, with the p-value provided above the line, Figure S5: (A) Group scan samples of three northern white-cheeked gibbons (Study ID 6) did not indicate differences in the group’s social and rest behavior as visitor abundance increased. A study of two female Sumatran orangutans (Study ID 8) detected increased interactive behavior and decreased rest behavior for both individuals when the orangutans and humans were in closest proximity, but social behavior did not show similar patterns based on human proximity. This finding is likely impacted by the differences between the two females in the percent of scans they spent in closest proximity to humans. Horizontal lines indicate a difference in behavior for an individual across the levels of the independent variable, with the p-value provided above the line, Figure S6: The fish in an interactive exhibit varied in their response to visitors: (A) As the number of visitors at the exhibit increased, the cownose ray decreased their social swimming and increased their solitary swimming (Study ID 9), but (B) only southern stingray and bonnethead sharks changed their behavior or location when there were visitors providing food provisions to the exhibit pool (Study ID 10). Horizontal lines indicate a difference in behavior for an individual across the levels of the independent variable, with the p-value provided above the line. Results from post-hoc Bonferroni tests are noted by letters above the bars; different letters indicate a pairwise differences at an alpha-level of 0.008.

## Appendix A. Methods for the 10 Independent Studies

There were 10 independent studies on 16 species at the Memphis Zoo during the months of September and October in the period 2012–2018. The methods for each study are detailed below, and they provide additional context to what is stated in the manuscript’s main text and Table 1, Table 2 and Table 3. All studies were designed to test the hypothesis that humans impact the behavior of zoo animals. Some studies originally had additional objectives (e.g., determining an overall activity budget for the species), but for the purpose of clarity, only the methods associated with testing the impact of the human-related variable (Table 2) on the behavior of zoo animals (Table 3) are presented. Analyses were not completed for behaviors that occurred for less than 5% of the behavioral scans. Behaviors that were not frequently exhibited were grouped in the behavioral category “other” in all studies. The order of the studies listed below follows the order presented in the main text of the manuscript (Table 1).

The independent variables associated with humans are defined in the main text (Table 2). The categorization for visitor abundance (0, 1–4, 5–9, and ≥10 visitors) remained consistent across the mammal studies. However, some exhibits did not experience heavy visitor traffic (≥10 visitors during a scan), and some exhibits always had at least one visitor present during a scan. When such situations arose, there were three comparison groups for visitor abundance instead of four comparison groups. The exhibits also varied in the extent to which the human observer recording data could be distant from the exhibit and still record data. When the human observer was unable to record data from a distance, their presence was counted as an individual because at times the zoo animals interacted with the human observer who collected data. All data were collected by people who were novel to the zoo animals.

All studies used chi-square tests to test whether behavior changed at different levels of the human-related independent variable (Table 2). We fully acknowledge the limitations of such analyses. We chose these analyses based on the work of previous studies [17,18], and based on the extent of our data. To minimize confounding variables across the 10 studies and 16 species, we analyzed each study independently. Furthermore, whenever possible, we tested if a behavioral change occurred for each individual animal studied. Given the importance of understanding individual responses [58,59,84], we did not want to pool data into an analysis and lose the individual component. We also tried to minimize bias in the representation of the data: not all size classes for visitor abundance were equally represented in all studies, for example. However, these unequal representations reflect the actual real-world differences in visitor presence and engagement at the different zoo exhibits. Therefore, we standardized these scans by calculating the percent of scans that each behavior was represented under each value of the independent variable. Our statistical analyses were based on our study’s goals of studying behavioral responses of individual animals, while trying to limit biases and misinterpretations of results. For each analysis, we tested if there was a change in an individual’s behavior (Table 3) based on changes in the value of the independent variable (Table 2). Because some behaviors were not exhibited by some animals, the number of analyses per individual varied. The independent variable(s) and behavior(s) tested for each animal is outlined below, based on each of the 10 independent studies. Overall, the critical p-value was 0.05 for statistical analyses, and therefore a behavioral change was noted when *p* ≤ 0.05 if analysis of one independent variable was undertaken. When we performed multiple analyses on a single individual (e.g., tested more than one independent variable), we used Bonferroni-corrected p-values to determine whether there were behavioral changes. When a difference in a particular behavior was detected for an independent variable, we followed up with a Bonferroni-adjusted post-hoc test based on the number of comparisons made. Details regarding data collection and analysis are described below for each of the 10 studies.

### Appendix A.1. Study ID 1: Gray Wolf

The study was conducted in September and October 2018 on four grey wolves (*Canis lupus*) in the Teton Trek exhibit at the Memphis Zoo. The four wolves (females: Meeka, Rocki, and Shiloh; male: Dakota) were siblings. At the time of the study, the four wolves had been in the exhibit for nine years. The wolf exhibit consisted of an inclined, elevated boardwalk, where zoo visitors walked to see the wolves below. Although there was vertical distance between the humans and wolves, there were no visual or audio barriers between zoo visitors and the wolves.

The behavior (Table A1) of each individual wolf was recorded using scan sampling at two-minute intervals, 120 min per session, for four sessions (248 scans for each wolf). With each scan, visitor abundance and noise were recorded. Visitor noise was categorized in one of three categories: low (visitor behaviors included walking, talking softly or not at all, and/or pushing a stroller), medium (visitor behaviors included “low” behaviors as well as running and/or talking loudly), and high (visitor behaviors included “medium” behaviors and playing loud music, yelling to another person or to the wolves, and/or howling). Ambient noise not created by the visitors was not recorded. Visitors were present during 63.2% of the scans. Of the total scans, visitor noise levels were categorized as none (no visitors: 36.8% of scans), low (53.2% of scans), medium (8.0%), and high (2.0%). The number of visitors per scan averaged 2.95 (range: 0 to 14).

**Table A1:** Wolf ethogram, with behaviors of focus indicated with an asterisk (*).

Behavior	Description
Alert *	
Rest with Head Up ^1^	Resting with head in observant, upward position
Stand ^1^	Standing still on all four legs, looking in one direction
Walk ^1^	Calm movement around exhibit, looking in one direction
Run ^1^	Quick movement around exhibit, looking in one direction
Stalk ^1^	Creeping movements in pursuit of another wolf or visitor
Vocalization ^1^	Howl, growl, or whimper
Rest *	Full body (including head) in recumbent position
Other	All other behavior

^1^ Categorized as an “alert” behavior, with all instances summed for analysis.

The human variables of focus were visitor abundance and visitor noise (Table 2). The zoo animal behaviors of focus were alert and rest. Behavioral data were summarized for each wolf individually. Chi-square tests were used to determine whether alert and rest behaviors differed as visitor abundance changed (0, 1–4, 5–9, and ≥10 visitors). Then, chi-square tests were used to determine whether there was a difference in the percent of scans an individual wolf exhibited alert behavior across the three classes of visitor noises. Each wolf and behavior were tested separately, for each of the two independent variables (visitor abundance and visitor noise). With two independent variables tested, we used an alpha level of 0.025 to determine when a difference in behavior occurred. When a behavioral difference was noted, we followed up with post-hoc Bonferroni adjustments with the alpha level set at 0.0083 for tests of the independent variable visitor abundance (as there were six pairwise comparisons) and an alpha level of 0.017 for tests of the independent variable visitor noise (three pairwise comparisons).

### Appendix A.2. Study ID 2: Cheetah

The study was conducted in September and October 2015 on two cheetahs (*Acinonyx jubatus*) in the cheetah exhibit in Cat Country at the Memphis Zoo. The two cheetahs (males: Kindu and Kasai) were siblings. At the time of the study, both cheetahs were 5 years old and had been at the Memphis Zoo for almost 3.5 years. The cheetah exhibit allowed for zoo visitors to walk approximately one-third of the perimeter of the exhibit and view the cheetahs from a concrete path. A moat and vegetation separated the zoo visitors from the exhibit. In this area, there were not visual or audio barriers between zoo visitors and the cheetahs.

Data were recorded using scan sampling at one-minute intervals, 120 min per session, for five sessions (605 scans for each cheetah). At each scan, the behavior (Table A2) of each cheetah and the number visitors in front of the exhibit were recorded. When the behavior was recorded, it was also noted whether or not the animal was demonstrating vigilance with the behavior. Visitor abundance was classified in four categories: 0 visitors, 1–4 visitors, 5–9 visitors, and ≥10 visitors. Zoo visitors were present in 73.4% of the 605 scans for each of the two cheetahs. Visitor abundance averaged 2.76 (range: 0 to 40 individuals) per scan.

**Table A2:** Cheetah ethogram, with behaviors of focus indicated with an asterisk (*).

Behavior	Description
Vigilance *	Prolonged stare at a specific location
Aggression *	Agonistic interactions between individuals through physical contact
Stereotypic *	Pacing: constant, repetitive walking back and forth, returning to the original position
Rest *	Recumbent or sitting without movement
Movement	Walking, trotting, or running
Other	All other behaviors not noted above

Data were analyzed for each cheetah individually. The human variable of focus was visitor abundance (Table 2). The zoo animal behaviors of focus were vigilance, aggression, stereotypic, and rest (Table A2). The percent of scans each cheetah engaged in each behavior was calculated at each of the four levels of visitor abundance. Because stereotypic pacing and aggressive behavior were not exhibited often by the cheetahs (<5% of the behavioral scans), these two behaviors were not analyzed based on visitor abundance. Chi-square tests were used to test whether there was a difference in each behavior of focus (vigilance and rest) at the four levels of visitor abundance for each individual cheetah. With one independent variable tested (visitor abundance), we used an alpha level of 0.05 to determine when a difference in behavior occurred. When a behavioral difference was noted, we followed up with post-hoc Bonferroni adjustments with the alpha level set at 0.0083 for tests of the independent variable visitor abundance (as there were six pairwise comparisons).

### Appendix A.3. Study ID 3: Lion

The study was conducted in September and October 2015 on three lions (*Panthera leo*) in lion exhibit in Cat Country at the Memphis Zoo. The three lions (male: 6.5-year-old Thabo; female littermates: 6.75-year-old Akeelah and Jamela) had all been at the Memphis Zoo for four years at the time of the study. The lion exhibit allowed for zoo visitors to walk approximately one-third of the perimeter of the exhibit and view the lions from a concrete path in the front of the exhibit, and from a small side opening. A moat and vegetation separated the zoo visitors from the exhibit. In the main front viewing area, there were not visual or audio barriers between zoo visitors and the lions.

Data were recorded using scan sampling at two-minute intervals, 120 min per session, for five sessions (310 scans for each lion). At each scan, the behavior (Table A3) of each lion and the number visitors in front of the exhibit and at the side of the exhibit were recorded. Vigilance was defined as the individual engaged in eye contact with a visitor or a prolonged stare at a location, and included the behaviors scan, approach, track and follow (Table A3).

**Table A3:** Lion ethogram, with behaviors of focus indicated with an asterisk (*).

Behavior	Description
Vigilance *	
Scan ^1^	Glance around exhibit without movement around exhibit
Approach ^1^	Moving toward a specific target
Track ^1^	Following a human with eyes or head; included staring at subject
Follow ^1^	Walking in same direction as target while displaying tracking behaviors
Rest *	Recumbent or sitting without movement
Walk	Moving around exhibit
Other	Activities not included above

^1^ Behaviors as “vigilance” behavior, with all instances summed for analysis.

Data were analyzed for each lion individually. The human variable of focus was visitor abundance. The zoo animal behaviors of focus were vigilance and rest (Table A3). Visitor abundance was classified in four categories: 0 visitors, 1–4 visitors, 5–9 visitors, and ≥10 visitors. Because zoo visitors were present in 100% of the scans, there were no data for the first category denoting no visitors. The number of visitors per scan averaged 7.76 (range: 2 to 39), with an average of 6.60 visitors in front of the exhibit (range: 2 to 33), and 1.56 on the side of the exhibit (range: 0 to 15). There were no visitors at the small side window for 63.9% of the scans. Behavioral patterns in resting and vigilance did not differ when visitor abundance was categorized based on total visitors versus visitors at the front of the exhibit for all lions (*p* > 0.05 for all), so analyses presented represent visitor abundance in total.

The percent of scans each lion engaged in each behavior of focus was calculated at each of the three levels of visitor abundance (1–4 visitors, 5–9 visitors, and ≥10 visitors). Chi-square tests were used to test whether there was a difference in each behavior at the three levels of visitor abundance noted in the study. With one independent variable tested (visitor abundance), we used an alpha level of 0.05 to determine when a difference in behavior occurred. When a behavioral difference was noted, we followed up with post-hoc Bonferroni adjustments with the alpha level set at 0.017 for tests of the independent variable visitor abundance (as there were three pairwise comparisons).

### Appendix A.4. Study ID 4: Southern Tamandua and Garnett’s Greater Galago

The study was conducted in September and October 2015 on two tamanduas (*Tamandua tetradactyla*) and four galagos (*Otolemur garnettii*) in the Animals of the Night nocturnal house at the Memphis Zoo. The tamanduas (female of unknown age: Mary Anne; 3-year-old male: Mr. Wendall) were housed in the same exhibit. Two of the four galagos (1-year-old male siblings Yoda and Obi Wan) were in one exhibit together; the other two galagos were housed separately with other species (15-year-old male Jaymes with a skunk, *Mephitis mephitis*; 7-year-old male Chewbacca with a wombat, *Lasiorhinus latifrons*, and an armadillo, *Tolypeutes trininctus*). All exhibits were located indoors during data collection the lights were set to simulate night. Features (e.g., rock wall, trash can, den) allowed for the zoo animals to be on exhibit but not visible to the researchers. Visitors could view the animals in each of the exhibits from the front wall of the exhibit, which consisted of glass. It was possible for zoo visitors to approach the glass and touch it.

Data were recorded using scan sampling at one-minute intervals, 66 min per session, for five sessions (an average of 311 scans for each individual). At each scan, the behavior (Table A4) of each tamandua and each galago was recorded. Visitor proximity (close: at the exhibit’s glass window, medium: less than 1 m from the exhibit window, and far: 1–2 m from the exhibit window) was also noted during each scan.

**Table A4:** Tamandua and galago ethogram, with behaviors of focus indicated with an asterisk (*).

Behavior	Description
Interactive *	Approach the glass window, place appendages on window, or make direct eye contact with human observer
Social *	Interact with another individual through touch (e.g., grooming, touching bodies, copulation)
Stereotypic *	Repetitive walking back and forth, or sway body back and forth
Rest *	Recumbent or sitting without movement
Out of Sight *	Animal is on exhibit, but not visible to the researcher (behind or inside exhibit feature)
Movement	Move body from one location to another, in horizontal or vertical position (e.g., walk, climb, leap)
Eat/Drink	Consume food or water
Other	Activities not included above

Data were analyzed for each tamandua and galago individually. The human variable of focus was proximity. The zoo animal behaviors of focus were interactive, social, stereotypic, rest, and out of sight (Table A4). The percentage of scans the individual animal engaged in each behavior was noted when the visitor(s) were at three distances: close, medium, and far. Chi-square tests were used to test for differences in each behavior of interest for each individual tamandua and galago, if the behavior represented 5% or more of the individual animal’s scans. Therefore, we tested the following behaviors: interactive (four galagos), social (two galagos), stereotypic (one tamandua and two galagos), rest (four galagos), and out of sight (two tamanduas and four galagos). With one independent variable tested (visitor proximity), we used an alpha level of 0.05 to determine when a difference in behavior occurred. When a behavioral difference was noted, we followed up with post-hoc Bonferroni adjustments with the alpha level set at 0.017 for tests of the independent variable visitor proximity (as there were three pairwise comparisons).

### Appendix A.5. Study ID 5: Mona Monkey, Northern Night Monkey, Northern White-Cheeked Gibbon, Bonobo, and Western Lowland Gorilla

The study was conducted across four weeks in October 2012 on five species of primates: Mona monkey (*Cercopithecus mona*), northern night monkey (*Aotus trivirgatus*), northern white-cheeked gibbon (*Nomascus leucogenys*), bonobo (*Pan paniscus*), and western lowland gorilla (*Gorilla gorilla*). These five species were housed separately, and there were two exhibits of white-cheeked gibbons (Table A5).

**Table A5:** Primate species studied for project #5.

Species	Individuals ^1^	Relationship	Exhibit	Mean Visitor Abundance (Range)
Mona Monkey	Drew (male; 17 years old); Tiffany (female; 15 years old)	Siblings	Outdoor	2.64 (1–16)
Northern Night Monkey	Blossom (female; 23 years old); Bubbles (female; 8 years old); Buttercup (female; 7 years old)	Blossom mother to siblings Bubbles and Buttercup	Indoor in nocturnal house; glass window along length of exhibit	2.07 (1–7)
White-Cheeked Gibbon	Timmi (female; 23 years old); Donta (male; 19 years old)	None	Outdoor	9.38 (1–54)
White-Cheeked Gibbon	Ringo (male; 10 years old); Tallulah (9 years old)	Siblings	Outdoor; glass window along length of exhibit	9.54 (1–27)
Bonobo	Mofana (male; 34 years old); Lisala (female; 20 years old); Kiri (female; 19 years old); Lily (female; 15 years old); Sukari (female; 7 years old)	Kiri mother to Sukari; Lisala and Lily half-sisters	Indoor-Outdoor; glass window along length of exhibit	3.37 (1–15)
Western Lowland Gorilla	Mwelu (male; 26 years old); Kwizera (female; 24 years old); Penny (female; 23 years old); Kebara (female; 15 years old)	Penny mother to Kebara	Outdoor	10.10 (3–37)

^1^ Age at the time of the study.

Data were recorded using scan sampling at two-minute intervals, 40 min per session, for four sessions (81 scans for each individual). At each scan, the behavior (Table A6) of each primate and the number visitors in front of the exhibit were recorded.

**Table A6:** Primate ethogram, with behaviors of focus indicated with an asterisk (*).

Behavior	Description
Interactive *	Approach human via moving toward the glass window or exhibit edge, place appendages on window where human present, or make direct eye contact with human
Social *	Interact with another individual through touch (e.g., grooming, touching bodies, copulation)
Aggression *	Agonistic interactions between individuals through physical contact
Rest *	Recumbent or sitting without movement
Out of sight *	Animal is on exhibit, but not visible to the researcher (behind or inside exhibit feature)
Movement	Move body from one location to another, in horizontal or vertical position (e.g., walk, climb, leap)
Eat/Drink	Consume food or water
Other	Activities not included above

The human variable of focus was visitor abundance. The zoo animal behaviors of focus were interactive, social, aggression, rest, and out of sight (Table A6). Data were analyzed for each primate individually when identifications were consistently verified for the entire duration of the study. Individual identification occurred for all gibbons and the male gorilla. For these animals, the percent of scans each individual engaged in each behavior of focus was calculated at each of the levels of visitor abundance (1–4 visitors, 5–9 visitors, and ≥10 visitors); there was always a human present. For the northern night monkeys, only the categories of 1–4 visitors and 5–9 visitors were represented because visitor abundance never exceeded seven people during a scan. Chi-square tests were used to test whether there were differences across groups for each behavior of focus that comprised ≥ 5% of the behavioral scans for that individual. These behaviors were interactive, rest, and out of sight (Mona monkeys); social and rest (northern night monkeys); interaction (three gibbons), social (four gibbons), and rest (four gibbons); interactive, social, and rest (bonobos); and interactive and rest (gorillas). For the pair of unrelated gibbons, comparisons of behavior at low visitor abundance (1–4 visitors) were compared with higher visitor abundance (≥10 visitors) because there were only 9 scans in total when there were 5–9 visitors present. With one independent variable tested (visitor abundance), we used an alpha level of 0.05 to determine when a difference in behavior occurred. When a behavioral difference was noted, we followed up with post-hoc Bonferroni adjustments with the alpha level set at 0.017 for tests of the independent variable visitor abundance (as there were three pairwise comparisons). Pairwise comparisons were not made for the northern night monkeys and the pair of unrelated gibbons because there were only two categories of visitor abundance represented.

For animals that were not identified to individual week after week, data were pooled for the entire group, and the percent of scans that the group engaged in each behavior of focus were calculated and tested using chi-square tests. Individual identifications would have been ideal, but given the number of individuals studied (17 individuals), it was not possible to confirm individual identifications at all times for some of the species. Because the bonobos on exhibit varied weekly, data were pooled to have an adequate sample size.

### Appendix A.6. Study ID 6: Northern White-Cheeked Gibbons

The study was conducted in September and October 2010 on three northern white-cheeked gibbons (*Nomascus leucogenys*) in the China exhibit area at the Memphis Zoo. The three gibbons (17-year-old male and 21-year-old female Timmi with their 10-year-old son Jing Chi) had all been at the Memphis Zoo for 6 years at the time of the study. The gibbon exhibit allowed for zoo visitors to walk approximately 45% of the perimeter of the exhibit, and view the gibbons from a concrete path. A water body separated visitors from the gibbons. There were not visual or audio barriers between zoo visitors and the gibbons.

Data were recorded using scan sampling at two-minute intervals, 60 min per session, for nine sessions (270 scans for each gibbon). At each scan, the behavior (Table A7) of each gibbon and the number visitors at the exhibit were recorded. Visitor abundance was classified in four categories: 0 visitors, 1–4 visitors, 5–9 visitors, and ≥10 visitors. The number of visitors per scan averaged 7.09 (range: 0 to 47).

**Table A7:** Gibbon ethogram, with behaviors of focus indicated with an asterisk (*).

Behavior	Description
Social *	Interact with another individual through touch (e.g., grooming, touching bodies, copulation)
Rest*	Recumbent or sitting without movement
Movement	Move body from one location to another, in horizontal or vertical position (e.g., walk, climb, leap)
Eat/Drink	Consume food or water
Other	Activities not included above

Data were analyzed for the three gibbons combined because the researchers did not always confirm identify for the two males. The human variable of focus was visitor abundance. The zoo animal behaviors of focus were social and rest. The percent of scans the gibbons engaged in rest behavior was calculated at each of the four levels of visitor abundance (0 visitors, 1–4 visitors, 5–9 visitors, and ≥10 visitors). The same calculations were completed for social behavior. Chi-square tests were used to test whether there were differences in social and rest behavior at the four levels of visitor abundance. With one independent variable tested (visitor abundance), we used an alpha level of 0.05 to determine when a difference in behavior occurred. If a behavioral difference was noted, we followed up with post-hoc Bonferroni adjustments with the alpha level set at 0.0083 for tests of the independent variable visitor abundance (as there would be six pairwise comparisons).

### Appendix A.7. Study ID 7: Northern White-Cheeked Gibbon

The study was conducted in September and October 2014 on two northern white-cheeked gibbons (*Nomascus leucogenys*) at the Cat House Café exhibit at the Memphis Zoo. The two gibbons (12-year-old male Ringo and 11-year-old female Talulah) had been at the Memphis Zoo since birth. The gibbon exhibit has two of its four sides available to zoo visitors; the front area is a small viewing area where visitors are separated from the gibbons by a dry moat and vegetation. The length of one side of the exhibit is a set of large windows, where people from inside the Cat House Café can view the gibbons. Gibbons and humans can touch the windows from their respective sides. There were no visual barriers between zoo visitors and the gibbons. While there were no audio barriers in the small front viewing area, the windows reduced the audio between humans in the café and the gibbons; however, visitors did knock on the glass windows at times.

Data were recorded using scan sampling at two-minute intervals, 120 min per session, for four sessions (248 scans for each gibbon). At each scan, the behavior (Table A8) of each gibbon and whether or not visitors were present were recorded. If visitors were present, it was noted if the visitors were initiating contact (e.g., touching or tapping the windows). Further, at each scan, the location of each gibbon was noted (e.g., window, grass, climbing structure, rocks).

**Table A8:** Gibbon ethogram, with behaviors of focus indicated with an asterisk (*).

Behavior	Description
Interactive *	Approach human via moving toward the glass window or exhibit edge, place appendages on window where human present, or make direct eye contact with human
Social *	Interact with another individual through touch (e.g., grooming, touching bodies, copulation)
Rest *	Recumbent or sitting without movement
Movement	Move body from one location to another, in horizontal or vertical position (e.g., walk, climb, leap)
Eat/Drink	Consume food or water
Other	Activities not included above

Data were analyzed for each individual gibbon. The human variables of focus were visitor presence and visitor soliciting interactions. The zoo animal behaviors of focus were interactive, social, and rest (Table A8), as well as location at the window. Chi-square tests were used to test whether there was a change in each behavior of focus, as well as the location of the gibbon at the viewing window, based on (1) the presence/absence of visitors and (2) when visitors were present, if the visitors were engaged in visitor-initiated behaviors. With two independent variables tested (visitor presence and visitor soliciting interactions), we used an alpha level of 0.025 to determine when a difference in behavior occurred. Because all comparisons were between two levels of the independent variable (e.g., visitors present vs. visitors absent; visitors solicited an interaction vs. visitors did not solicit an interaction), no post-hoc tests were conducted on these data.

### Appendix A.8. Study ID 8: Sumatran Orangutan

The study was conducted in September and October 2012 on three Sumatran orangutans (*Pongo abelii*) at the Memphis Zoo. The three orangutans (females: 34-year-old Chickie and 14-year-old Jahe; male: 30-year-old Tombak) had been at the Memphis Zoo for 24, 2, and 18 years, respectively. The orangutan exhibit had approximately one-third of its perimeter available to zoo visitors. Visitors were separated from the gibbons by a dry moat and vegetation; there were no visual or audio barriers.

The purpose of this project was to determine whether there was a relationship between the physical location of the human observer and (1) the location of the orangutan and (2) the behavior of the orangutan. The exhibit was divided into nine sections (front of the exhibit: left, right, center; middle of the exhibit: left, right, center; and back of the exhibit: left, right, center). The front of the exhibit was in closest proximity to where humans were located. The human’s observation area was divided into thirds (corresponding to the front left, front center, and front right sections of the orangutan’s exhibit), and the human observer moved to one of the three locations every 20 min. Data were recorded using scan sampling at two-minute intervals, 120 min per session, for four sessions (248 scans for each orangutan). At each scan, the behavior (Table A9) of each orangutan was recorded, as well as the position of the orangutan and the human observer. The closest proximity between the human observer and orangutan occurred when the human and orangutan occupied the same corresponding section of the exhibit or viewing area: for example, the human was located in the front-right section and the orangutan was located in the front-right section of the exhibit.

**Table A9:** Orangutan ethogram, with behaviors of focus indicated with an asterisk (*).

Behavior	Description
Interactive *	Make eye contact with human; included holding out hand and uncurling fingers to human observer
Social *	Interact with another individual through touch (e.g., grooming, touching bodies, copulation)
Aggression *	Agonistic interactions between individuals through physical contact
Rest *	Recumbent or sitting without movement
Movement	Move body from one location to another, in horizontal or vertical position (e.g., walk, climb, leap)
Eat/Drink	Consume food or water
Other	Activities not included above

Data were analyzed for each individual orangutan. Although there were three orangutans total in the group, the male was on exhibit for only one of the four data sessions, and he spent 100% of the scans in the same location, so he was excluded from the analysis. The human variable of focus was proximity. The zoo animal behaviors of focus were location, interactive, social, aggression, and rest (Table A9). A chi-square test was used for each of the two female orangutans to test whether there was a relationship between the human observer’s location and the orangutan’s location. Then, chi-square tests were used to test whether there was a difference in the behaviors of focus when the orangutan was in closest proximity with the human observer versus when the orangutan was at further proximity from the human observer. Specifically, the following behaviors were analyzed because they each represented at least 5% of the scans: interactive, social, and rest. With two levels of analysis with the proximity variable, we used an alpha level of 0.025 in the statistical tests. Because all comparisons were between two levels of the independent variable (e.g., orangutan and human were in closest proximity: yes or no), no post-hoc tests were conducted on these data.

### Appendix A.9. Study ID 9: Cownose Ray

The study was conducted in September and October 2013 on cownose stingrays (*Rhinoptera bonasus*) at the Stingray Bay seasonal exhibit at the Memphis Zoo. At the time of the study there were 12 males and 13 females; three of these females were pregnant. Also in the exhibit were southern stingrays (*Hypanus americanus*), bonnethead sharks (*Sphyrna tiburo*), brownbanded bamboo sharks *Chiloscyllium punctatum* and white-spotted bamboo sharks *Chiloscyllium plagiosum*. This study focused only on the cownose stingrays.

The 17,000-gallon saltwater pool was approximately 45 cm deep throughout the study area, and there was an oxygenating waterfall at the southern end of the pool. Zoo visitors could stand along the entire perimeter of the pool, and reach into the water to interact with the stingrays and sharks. Food (fish) was available for purchase, and guests could feed these fish to the animals in the pool. The maximum number of interactive visitors during a scan was 30.

One of the objectives of this study was to determine whether human interactions impacted the social behavior of the cownose stingrays. Data were recorded using group scan sampling at two-minute intervals for 60 scans for each of the four research sessions (240 scans in total). At each scan, the behavior (Table A10) of each cownose stingray was recorded, as well as the number of zoo visitors along the perimeter of the exhibit.

**Table A10:** Cownose ethogram, with behaviors of focus indicated with an asterisk (*).

Behavior	Description
Social swim *^,1^	Swim in same direction within two body lengths of one or more other cownose stingray.
Solitary swim *^,1^	Swim more than two body lengths from another cownose stingray, or swim in the opposite direction of the other stingray.
Social rest *^,1^	No movement; individual is at the bottom of the pool within two body lengths from another resting conspecific.
Solitary rest *^,1^	No movement; individual is at the bottom of the pool more than two body lengths from another resting conspecific.

^1^ Solitary swim and rest were grouped together as solitary behavior, while social swim and rest were grouped together as social behavior for the analyses.

Data were analyzed for the combined group scans, as the identities of the individual cownose stingray could not be confirmed. The human variable of focus was visitor abundance. Visitor abundance was classified into four categories: 0, 1–10, 11-20, and ≥21 visitors. The zoo animal behaviors of focus were social and solitary swim, and social and solitary rest (Table A10). A chi-square test was used to determine whether the percent of scans that the cownose stingray engaged in social swim behavior versus solitary swim behavior changed as interactive visitor abundance changed; social and solitary rest behavior occurred for <5% of the scans, so these behaviors were not analyzed. With one independent variable tested (visitor abundance), we used an alpha level of 0.05 to determine when a difference in swimming occurred. When a behavioral difference was noted, we followed up with post-hoc Bonferroni adjustments with the alpha level set at 0.0083 for tests of the independent variable visitor abundance (as there were six pairwise comparisons).

### Appendix A.10. Study ID 10: Cownose Ray, Southern Stingray, Bonnethead Shark, Brownbanded Bamboo Shark, and White-Spotted Bamboo Shark

The study was conducted in September and October 2015 on 38 cownose stingrays (*Rhinoptera bonasus*), 6 southern stingrays (*Hypanus americanus*), 4 bonnethead sharks (*Sphyrna tiburo*), 2 brownbanded bamboo sharks (*Chiloscyllium punctatum*), and 16 white-spotted bamboo sharks (*Chiloscyllium plagiosum*) at the Stingray Bay seasonal exhibit at the Memphis Zoo. This study focused on the behaviors of all five species of fish in the pool.

The 17,000-gallon saltwater pool was approximately 45 cm deep throughout the study area, and there was an oxygenating waterfall at the southern end of the pool. Zoo visitors could stand along the entire perimeter of the pool, and reach into the water to interact with the stingrays and sharks. Food (fish) was available for purchase, and guests could feed these fish to the animals in the pool. Such feeding occurred for 33% of the scans.

One of the objectives of this study was to determine whether feeding by zoo visitors impacted the behavior of the stingrays and sharks. Data were recorded using group scan sampling, as individual identifications were not possible. At each scan, the behavior (Table A11) of each individual in sight was recorded, as well as whether there was food provided by the zoo visitors. Data were collected for one of three animal categories (cownose stingray, southern stingray, and sharks) at a time. Doing so allowed for better tracking of the 66 individuals in the study. Data on the sharks were collected together, but tallied separately between the diurnal bonnethead shark and nocturnal bamboo sharks. Each animal grouping (cownose stingray, southern stingray, bonnethead shark, or bamboo shark) had 70 (stingrays) or 65 (sharks) independent events recorded. Feeding by zoo visitors occurred for 33% of events for the cownose rays and southern rays and 34% of the events for the sharks during the entire study. Each research session lasted 120 min, and there were 5 session days in total. Due to the size of the pool, the large number of cownose rays, and the limited mobility of the sharks during the study, the researcher moved between two locations on either side of the pool to account for any bias in detection of the species or particular individuals. Upon moving to the other location, the researcher waited three minutes before starting data collection.

**Table A11:** Stingray and shark ethogram, with behaviors of focus indicated with an asterisk (*).

Behavior ^1^	Description
Swim, perimeter *	Forward movement within 1 m of the pool’s perimeter.
Swim, inner *	Forward movement more than 1 m from the pool’s perimeter.
Swim, enrichment *	Forward movement within or on top of an enrichment item in the pool.
Rest, perimeter *	No movement; individual is at the bottom of the pool, within 1 m of the pool’s perimeter.
Rest, inner *	No movement; individual is at the bottom of the pool, more than 1 m from the pool’s perimeter.
Rest, enrichment *	No movement; individual is within or on top of an enrichment item in the pool.

^1^ The behaviors were coded as swim, rest, or enrichment; swim or rest; and inner or perimeter of pool.

Data were analyzed for each of the four groups separately (cownose stingray, southern stingray, bonnethead shark, and the two species of bamboo sharks). The human variable of focus was food provisioning. The zoo animal behaviors of focus were swim, enrichment, and rest—with these behaviors noted as occurring along the periphery of the exhibit (perimeter) and the inner part of the exhibit (Table A11). Chi-square tests were used to determine whether there were differences in rest, swim, and location based on the presence of food provisioning by visitors. In addition, for the nocturnal bamboo sharks, a chi-square test was used to test whether interaction with enrichment differed based on the presence of food provisioning by visitors; the other three species did not interact with enrichment enough for statistical analysis to be conducted. With one independent variable tested (food provisioning: yes or no), we used an alpha level of 0.05 to determine when a difference in swimming occurred and location occurred. Because all comparisons were between two levels of the independent variable (food: yes or no), no post-hoc tests were conducted on these data.
