# Supplementary material for: Widespread Behavioral Responses by Mammals and Fish to Zoo Visitors Highlight Differences between Individual Animals
Source: Animals (Basel). 2020 Nov 13;10(11):2108. doi: 10.3390/ani10112108 (PMC7697811; doi:10.3390/ani10112108)
Supplement: Supplementary file 1 [file animals-10-02108-s001.pdf]

animals

MDPI

Supplementary Materials: Widespread Behavioral Responses by Mammals and Fish to Zoo Visitors Highlight Differences between Individual Animals

Table S1. Full summary of results, listed in order by Study ID [1]. All zoo animal behaviors of interest (Table 3) that occurred ≥5% of the scans are noted. Items in bold indicate a significant difference between comparison groups of the independent variable (human variable), based on the adjusted alpha-level from Bonferroni corrections. Where differences existed, the direction of patterns in the differences are noted. Results from pairwise comparisons indicated a direction of difference (Figures S1–S6).

Study ID: Species	Human Variable [2]	Zoo Animal Behavior [2]	Alpha-Level [3]	Results
1: Grey wolf (*Canis lupus*); *Figure* S1A-C	Abundance↑	Alert↑	0.025	**Shiloh: $X^2 = 14.64$, $df = 3$, $p = 0.0021$**<br>**Rocki: $X^2 = 25.479$, $df = 3$, $p<0.001$**
	Abundance	Alert (NS)	0.025	Dakota: $X^2 = 7.29$, $df = 3$, $p = 0.063$<br>Meeka: $X^2 = 2.44$, $df = 3$, $p = 0.49$
	Abundance↑	Rest (NCP)	0.025	**Dakota: $X^2 = 13.22$, $df = 3$, $p = 0.0042$**
	Abundance	Rest (NS)	0.025	Shiloh: $X^2 = 8.10$, $df = 3$, $p = 0.044$<br>Rocki: $X^2 = 3.85$, $df = 3$, $p = 0.28$<br>Meeka: $X^2 = 1.02$, $df = 3$, $p = 0.80$
	Noise level↑	Alert↑	0.025	**Dakota: $X^2 = 27.70$, $df = 2$, $p<0.001$**<br>**Meeka: $X^2 = 20.88$, $df = 2$, $p<0.001$**
	Noise level↑	Alert (NCP)	0.025	**Shiloh: $X^2 = 15.69$, $df = 2$, $p<0.001$**
	Noise level	Alert (NS)	0.025	Rocki: $X^2 = 1.85$, $df = 2$, $p = 0.39$
2: Cheetah (*Acinonyx jubatus*); *Figure* S1D-E	Abundance↑	Vigilance↑	0.05	**Kindu: $X^2 = 13.55$, $df = 3$, $p = 0.0036$**<br>**Kasai: $X^2 = 17.87$, $df = 3$, $p<0.001$**
	Abundance	Rest (NS)	0.05	Kindu: $X^2 = 0.84$, $df = 3$, $p = 0.84$<br>Kasai: $X^2 = 2.26$, $df = 3$, $p = 0.52$
3: Lion (*Panthera leo*); *Figure* S1F-G	Abundance↑	Vigilance↑	0.05	**Thabo: $X^2 = 36.17$, $df = 2$, $p<0.001$**<br>**Akeelah: $X^2 = 10.30$, $df = 2$; $p = 0.0058$**<br>**Jamela: $X^2 = 6.16$, $df = 2$, $p = 0.046$**
	Abundance↑	Rest↓	0.05	**Thabo: $X^2 = 10.62$, $df = 2$, $p = 0.0049$**<br>**Akeelah: $X^2 = 10.15$, $df = 2$, $p = 0.0063$**<br>**Jamela: $X^2 = 7.76$, $df = 2$, $p = 0.021$**
4: Southern tamandua (*Tamandua tetradactyla*); *Figure* S2	Proximity	Stereotypic (NS)	0.05	Mr. Wendell: $X^2 = 0.54$, $df = 2$, $p = 0.76$
	Proximity	Out of sight (NS)	0.05	Mr. Wendell: $X^2 = 2.32$, $df = 2$, $p = 0.31$<br>Mary Anne: $X^2 = 3.10$, $df = 2$, $p = 0.21$
4: Garnett's greater galago (*Otolemur garnettii*); *Figure* S2	Proximity: Close	Interactive↑	0.05	**Obi Wan: $X^2 = 8.00$, $df = 2$, $p = 0.018$**
	Proximity	Interactive (NS)	0.05	Yoda: $X^2 = 3.71$, $df = 2$, $p = 0.16$<br>Jaymes: $X^2 = 5.19$, $df = 2$, $p = 0.074$<br>Chewbaca: $X^2 = 2.71$, $df = 2$, $p = 0.26$
	Proximity	Social (NS)	0.05	Obi Wan: $X^2 = 1.50$, $df = 2$, $p = 0.47$<br>Yoda: $X^2 = 0.36$, $df = 2$, $p = 0.83$
	Proximity: Close	Stereotypic↑	0.05	**Yoda: $X^2 = 7.69$, $df = 2$, $p = 0.021$**
	Proximity: Close	Rest↓	0.05	**Yoda: $X^2 = 9.84$, $df = 2$, $p = 0.0073$**<br>**Chewbaca: $X^2 = 12.84$, $df = 2$, $p = 0.0016$**
	Proximity	Rest (NS)	0.05	Obi Wan: $X^2 = 1.44$, $df = 2$, $p = 0.49$<br>Jaymes: $X^2 = 3.16$, $df = 2$, $p = 0.21$

	Proximity: Close	Out of sight↑	0.05	**Chewbaca: $X^2 = 9.64$, $df = 2$, $p = 0.0081$**
	Proximity	Out of sight (NS)	0.05	Yoda: $X^2 = 0.89$, $df = 2$, $p = 0.64$
	Proximity: Medium [4]	Stereotypic↑	0.05	**Jaymes: $X^2 = 31.67$, $df = 2$, $p<0.001$**
	Proximity: Medium	Out of sight↑	0.05	**Jaymes: $X^2 = 11.08$, $df = 2$, $p = 0.0039$**
	Proximity: Far	Out of sight↑	0.05	**Obi Wan: $X^2 = 21.49$, $df = 2$, $p<0.001$**
5: Mona monkey (*Cercopithecus mona*) [5]; *Figure* S3	Abundance↑	Interactive↑	0.05	**$X^2 = 66.01$, $df = 2$, $p<0.001$**
	Abundance↑	Rest↓	0.05	**$X^2 = 9.33$, $df = 2$, $p = 0.0094$**
	Abundance↑	Out of sight↓	0.05	**$X^2 = 35.71$, $df = 2$, $p<0.001$**
5: Northern night monkey (*Aotus trivirgatus*) [5]; *Figure* S3	Abundance↑	Social↑	0.05	**$X^2 = 13.192$, $df = 1$, $p<0.001$**
	Abundance↑	Rest↓	0.05	**$X^2 = 17.32$, $df = 1$, $p<0.001$**
5: Northern white-cheeked gibbon (*Nomascus leucogenys*) – traditional exhibit; *Figure* S3	Abundance↑	Interactive↑	0.05	**Donta: $X^2 = 9.23$, $df = 1$, $p = 0.0024$**
	Abundance↑	Social↓	0.05	**Donta: $X^2 = 7.42$, $df = 1$, $p = 0.0065$**<br>**Timmi: $X^2 = 14.80$, $df = 1$, $p<0.001$**
	Abundance↑	Rest↓	0.05	**Donta: $X^2 = 5.31$, $df = 1$, $p = 0.021$**<br>**Timmi: $X^2 = 15.48$, $df = 1$, $p<0.001$**
5: Northern white-cheeked gibbon (*Nomascus leucogenys*) – indirect interactions exhibit; *Figure* S3	Abundance↑	Interactive↑	0.05	**Ringo: $X^2 = 13.42$, $df = 1$, $p = 0.0012$**<br>**Tallulah: $X^2 = 12.32$, $df = 1$, $p = 0.0021$**
	Abundance↑	Social↓	0.05	**Ringo: $X^2 = 10.00$, $df = 1$, $p = 0.0016$**<br>**Tallulah: $X^2 = 9.003$, $df = 1$, $p = 0.011$**
	Abundance↑	Rest↓	0.05	**Ringo: $X^2 = 29.53$, $df = 1$, $p < 0.001$**<br>**Tallulah: $X^2 = 10.23$, $df = 1$, $p = 0.0060$**
5: Bonobo (*Pan paniscus*) [5]; *Figure* S3	Abundance	Interactive (NS)	0.05	$X^2 = 1.17$, $df = 2$, $p = 0.56$
	Abundance↑	Social↓	0.05	**$X^2 = 48.89$, $df = 2$, $p < 0.001$**
	Abundance↑	Rest↑	0.05	**$X^2 = 27.83$, $df = 2$, $p < 0.001$**
5: Western lowland gorilla (*Gorilla gorilla gorilla*) [5]; *Figure* S3	Abundance↑	Interactive↑	0.05	**Females: $X^2 = 6.98$, $df = 2$, $p = 0.030$**
	Abundance↑	Rest↓	0.05	**Females: $X^2 = 25.11$, $df = 2$, $p < 0.001$**
	Abundance	Rest (NS)	0.05	Male: $X^2 = 3.72$, $df = 2$, $p = 0.16$
	Abundance↓	Interactive↑	0.05	**Male: $X^2 = 6.40$, $df = 2$, $p = 0.041$**
6: Northern white-cheeked gibbon (*Nomascus leucogenys*) [5]; *Figure* S5A	Abundance↑	Social (NS)	0.05	$X^2 = 7.46$, $df = 3$, $p = 0.059$
	Abundance	Rest (NS)	0.05	$X^2 = 1.61$, $df = 3$, $p = 0.66$
7: Northern white-cheeked gibbon (*Nomascus leucogenys*); *Figure* S4	Presence: Yes	Interactive↑	0.025	**Ringo: $X^2 = 45.68$, $df = 1$, $p < 0.001$**<br>**Tallulah: $X^2 = 48.49$, $df = 1$, $p < 0.001$**
	Presence: Yes	Social↓	0.025	**Ringo: $X^2 = 5.01$, $df = 1$, $p = 0.025$**<br>**Tallulah: $X^2 = 5.42$, $df = 1$, $p = 0.020$**
	Presence: Yes	Rest↓	0.025	**Ringo: $X^2 = 5.33$, $df = 1$, $p = 0.021$**
	Presence: Yes	Rest (NS)	0.025	Tallulah: $X^2 = 3.81$, $df = 1$, $p = 0.051$
	Presence: Yes	Located at exhibit window↑	0.025	**Ringo: $X^2 = 44.97$, $df = 1$, $p < 0.001$**<br>**Tallulah: $X^2 = 47.91$, $df = 1$, $p < 0.001$**
	Solicit Interaction	Interactive (NS)	0.025	Ringo: $X^2 = 0.007$, $df = 1$, $p = 0.94$<br>Tallulah: $X^2 = 0.006$, $df = 1$, $p = 0.94$
	Solicit Interaction	Social (NS)	0.025	Ringo: $X^2 = 0.051$, $df = 1$, $p = 0.82$<br>Tallulah: $X^2 = 0.01$, $df = 1$, $p = 0.92$
	Solicit Interaction	Rest (NS)	0.025	Ringo: $X^2 = 1.11$, $df = 1$, $p = 0.29$<br>Tallulah: $X^2 = 0.016$, $df = 1$, $p = 0.90$

	Solicit Interaction	Located at exhibit window (NS)	0.025	Ringo: $X^2 = 0.42$, $df = 1$, $p = 0.52$<br>Tallulah: $X^2 = 0.31$, $df = 1$, $p = 0.58$
	Proximity: Close	Closest proximity	0.025	**Chickie: $X^2 = 21.91$, $df = 1$, $p < 0.001$**
	Proximity: Close	Proximity (NS)	0.025	Jahe: $X^2 = 3.39$, $df = 1$, $p = 0.066$
8: Sumatran orangutan (*Pongo abelii*); *Figure* S5B	Closest proximity	Interactive↑	0.025	**Chickie: $X^2 = 32.05$, $df = 1$, $p < 0.001$**<br>**Jahe: $X^2 = 11.89$, $df = 1$, $p < 0.001$**
	Closest proximity	Social↑	0.025	**Jahe: $X^2 = 5.05$, $df = 1$, $p = 0.025$**
	Closest proximity	Social (NS)	0.025	Chickie: $X^2 = 2.17$, $df = 1$, $p = 0.14$
	Closest proximity	Rest↓	0.025	**Chickie: $X^2 = 5.50$, $df = 1$, $p = 0.019$**<br>**Jahe: $X^2 = 16.91$, $df = 1$, $p < 0.001$**
9: Cownose ray (*Rhinoptera bonasus*) [5]; *Figure* S6A	Abundance↑	Social swim ↓	0.05	**$X^2 = 13.09$, $df = 3$, $p = 0.0045$**
	Abundance↑	Solitary swim ↑	0.05	**$X^2 = 17.48$, $df = 3$, $p < 0.001$**
10: Cownose ray (*Rhinoptera bonasus*) [5]; *Figure* S6B	Food provisioned	Swim (NS)	0.05	$X^2 = 0.066$, $df = 1$ $p = 0.42$
	Food provisioned	Periphery (NS)	0.05	$X^2 = 0.77$, $df = 1$ $p = 0.38$
10: Southern stingray (*Hypanus americanus*) [5]; *Figure* S6B	Food provisioned	Swim ↑	0.05	**$X^2 = 4.52$, $df = 1$ $p = 0.033$**
	Food provisioned	Periphery (NS)	0.05	$X^2 = 0.48$, $df = 1$ $p = 0.49$
10: Bonnethead shark (*Sphyrna tiburo*) [5]; *Figure* S6B	Food provisioned	Swim (NS)	0.05	$X^2 = 1.55$, $df = 1$ $p = 0.21$
	Food provisioned	Periphery ↑	0.05	**$X^2 = 14.54$, $df = 1$ $p < 0.001$**
10: Brownbanded (*Chiloscyllium punctatum*) and white-spotted (*Chiloscyllium plagiosum*) bamboo sharks [5]; *Figure* S6B	Food provisioned	Swim (NS)	0.05	$X^2 = 0.67$, $df = 1$ $p = 0.41$
	Food provisioned	Enrichment (NS)	0.05	$X^2 = 0.027$, $df = 1$ $p = 0.87$
	Food provisioned	Periphery (NS)	0.05	$X^2 = 0.38$, $df = 1$ $p = 0.54$

[1] Results including findings when there were behavioral differences based on the visitor variable, and when no behavioral differences were noted. The absence of a behavioral difference does not necessarily indicate that zoo visitors do not impact the behavior of the zoo animals. These results only depict whether a difference was noted in our studies. Details for each of the 10 studies are available in Appendix 1. [2] The human variables are defined in Table 2, while the zoo animal behavior is defined in Table 3. NS (not significant) indicates that there was no detected difference in behavior. NCP (no clear pattern) indicates that although there was a statistical difference in behavior for the independent variable tested, the pattern in behavioral change was not clearly apparent. [3] When multiple independent variables were tested, we adjusted the alpha level using Bonferroni corrections. The alpha level used is indicated because studies differed in the number of comparisons that were made. [4] This finding is the one instance when the mid-range value for the independent variable was the condition in which the behavioral response was statistically greater than low or high ranges of the independent variable. [5] Results represent patterns from group scan sampling, not individual scan sampling (except for gorillas, which were sampled by sex: one male and three females).

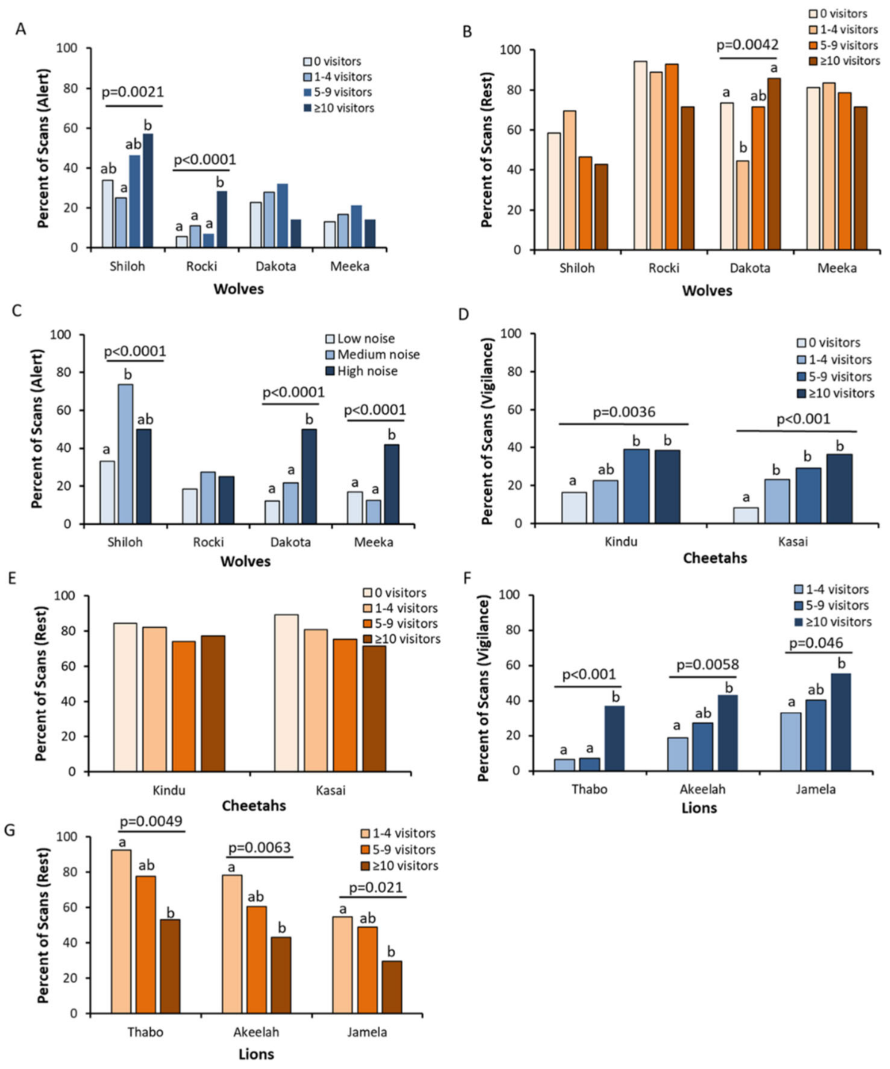

Figure S1. Individual wolves demonstrated varied responses to visitors: In some of the wolves, (A) alert and (B) rest behavior differed with changes in visitor abundance, and (C) three of the four wolves showed changes in alert behavior as visitor noise increased. Cheetahs (D) increased vigilance behavior at higher levels of visitor abundance, but rest behavior did not change. Lions (F) increased vigilance behavior and (G) decreased rest behavior at higher levels of visitor abundance. Horizontal lines indicate a difference in behavior for an individual across the levels of the independent variable, with the p-value provided above the line. Results from post-hoc Bonferroni tests are noted by letters above the bars; different letters indicate a pairwise differences at an alpha-level of 0.008 (A, B, D, and E) and 0.017 (C, F, and G). Data represent findings for three studies: wolves (Study ID 1), cheetahs (Study ID 2), and lions (Study ID 3).

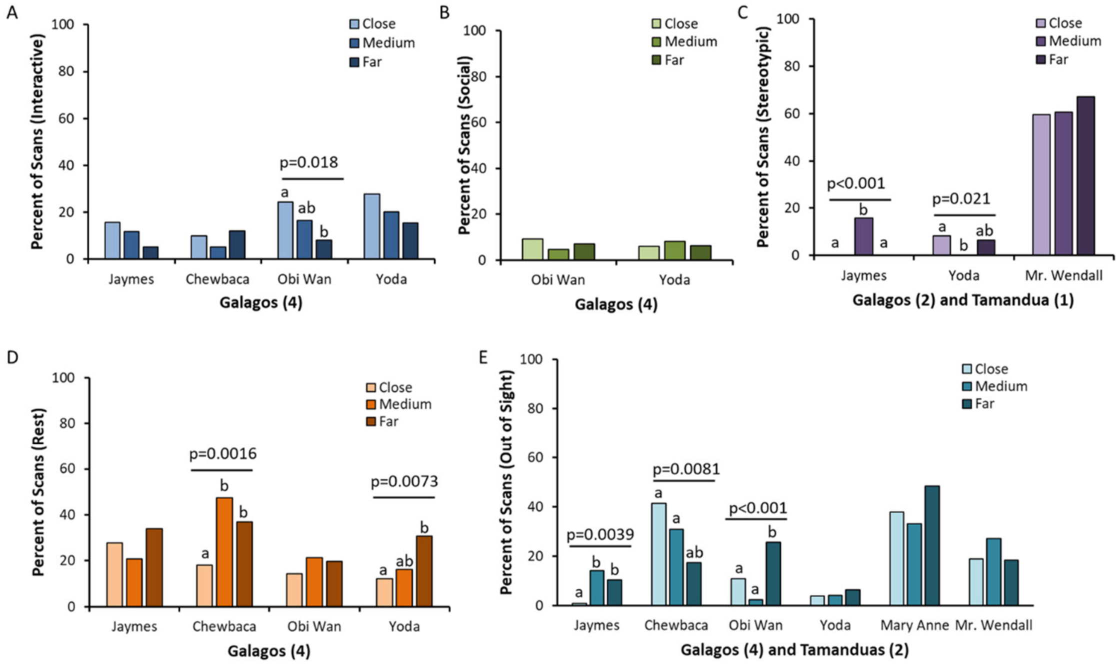

Figure S2. Increased proximity of humans to the exhibit was associated with (A) increased interactive behavior in one of four galagos; (B) no change in social behavior in two galagos; (C) inconsistent changes in stereotypic behavior in two galagos, while the male tamandua consistently exhibited high levels of stereotypic behavior; (D) decreased resting behavior for two of four galagos; and (E) inconsistent behavioral changes in three of four galagos, with no pattern of behavior change in the two tamanduas. Horizontal lines indicate a difference in behavior for an individual across the levels of the independent variable, with the p-value provided above the line. Results from post-hoc Bonferroni tests are noted by letters above the bars; different letters indicate a pairwise differences at an alpha-level of 0.017. Data represent findings for Study ID 4.

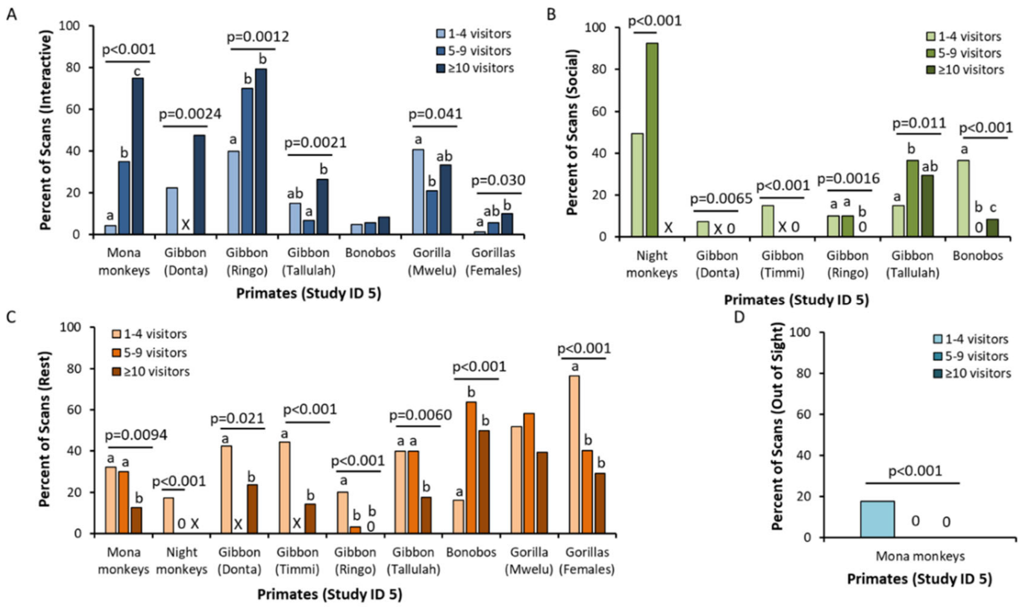

Figure S3. In one of the primate studies (Study ID 5), greater levels of visitor abundance were (A) associated with increased interactive behavior in the Mona monkeys, gibbons, and female gorillas; but (B) changes in social behavior did not follow a clear pattern across species. Increased visitor abundance was associated with (C) decreased rest in most of the primates, except for the bonobos and male gorilla, Mwelu. Only one species had more than 5% of scans out-of-sight, and such behavior was noted in the (D) Mona monkeys only when visitor abundance was low. An "X" indicates that the particular category for visitor abundance was not included in the analysis due to low sample size, while a "0" indicates that the behavior did not occur during the scans at that level of visitor abundance. There were always at least one visitor present for all scans, so there was no category of "0 visitors" in these analyses. Horizontal lines indicate a difference in behavior for an individual across the levels of the independent variable, with the p-value provided above the line. Results from post-hoc Bonferroni tests are noted by letters above the bars; different letters indicate a pairwise differences at an alpha-level of 0.017. Data represent findings for study ID 5.

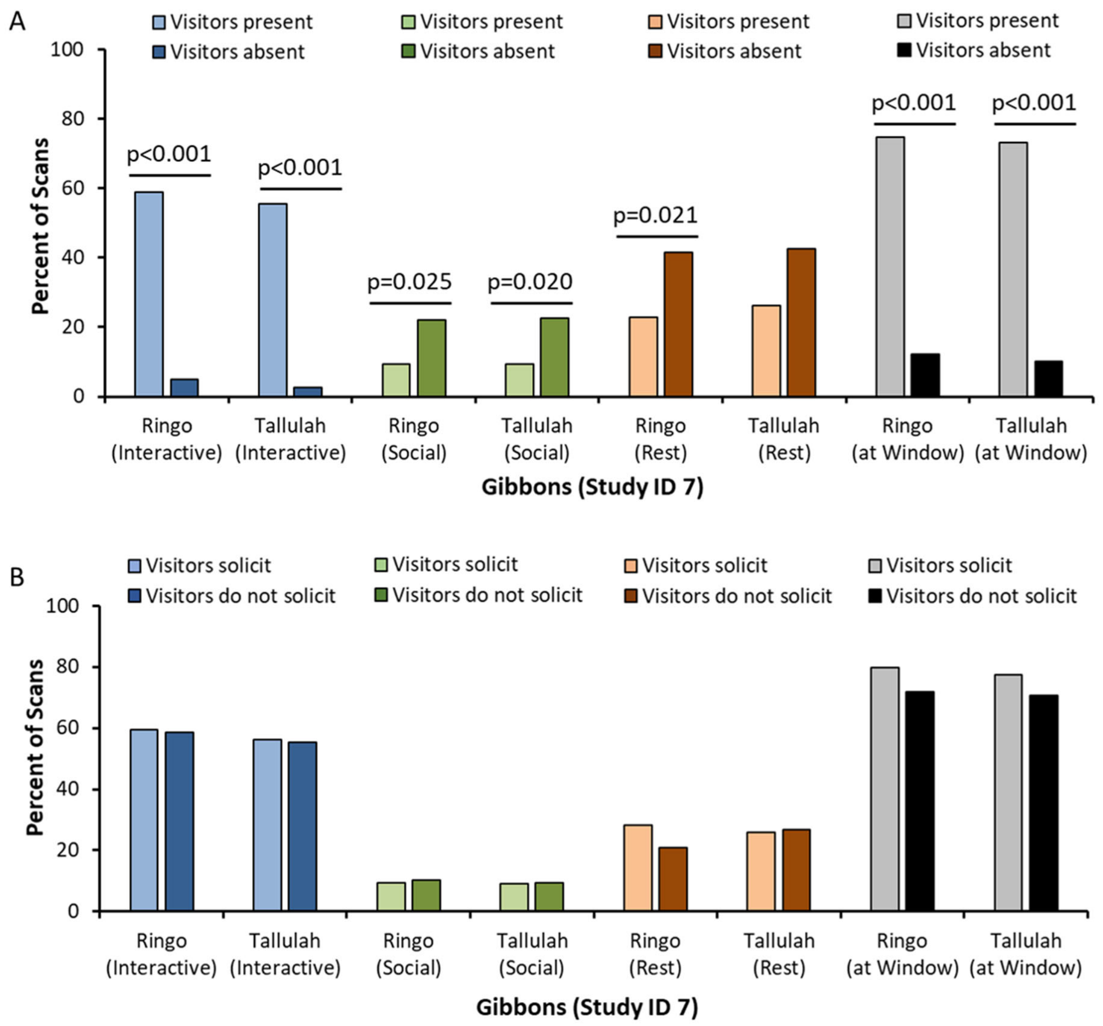

Figure S4. A sibling pair of northern white-cheeked gibbons was able to interact indirectly with zoo visitors via a long window that ran along the length of one side of the exhibit (Study ID 7). (A) When visitors were present, both the male and female gibbon exhibited increased interactive behavior, decreased social behavior with the other gibbon, and spent more time at the viewing window. The male also rested less when visitors were present. (B) When visitors were present at the window, there was no difference in behaviors by the gibbons when the visitors initiated contact with the gibbons versus when the visitors did not initiate contact. Horizontal lines indicate a difference in behavior for an individual across the levels of the independent variable, with the p-value provided above the line.

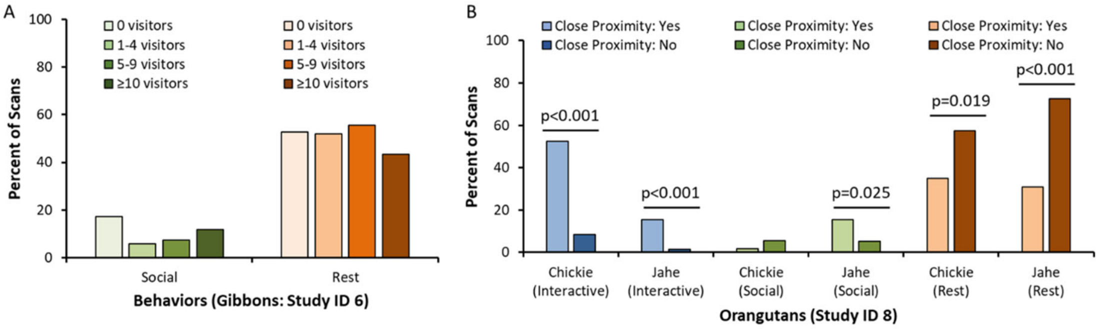

Figure S5. (A) Group scan samples of three northern white-cheeked gibbons (Study ID 6) did not indicate differences in the group's social and rest behavior as visitor abundance increased. A study of two female Sumatran orangutans (Study ID 8) detected increased interactive behavior and decreased rest behavior for both individuals when the orangutans and humans were in closest proximity, but social behavior did not show similar patterns based on human proximity. This finding is likely impacted by the differences between the two females in the percent of scans they spent in closest proximity to humans. Horizontal lines indicate a difference in behavior for an individual across the levels of the independent variable, with the p-value provided above the line.

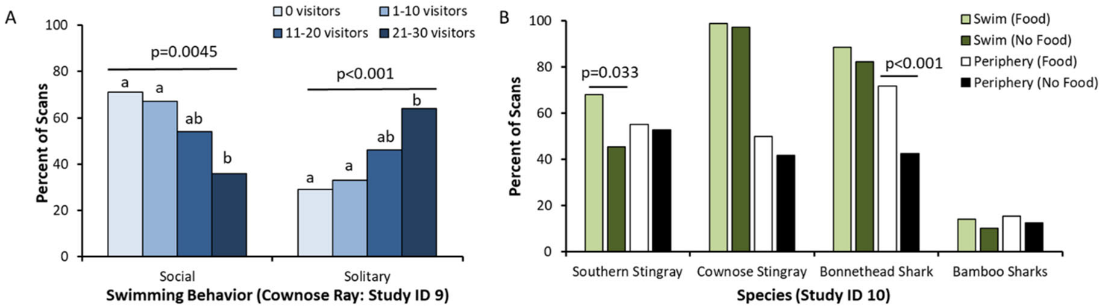

Figure S6. The fish in an interactive exhibit varied in their response to visitors: (A) As the number of visitors at the exhibit increased, the cownose ray decreased their social swimming and increased their solitary swimming (Study ID 9), but (B) only southern stingray and bonnethead sharks changed their behavior or location when there were visitors providing food provisions to the exhibit pool (Study ID 10). Horizontal lines indicate a difference in behavior for an individual across the levels of the independent variable, with the p-value provided above the line. Results from post-hoc Bonferroni tests are noted by letters above the bars; different letters indicate a pairwise differences at an alpha-level of 0.008.
